# Measured air quality impacts after teaching parents about cooking ventilation with a video: a pilot study

**DOI:** 10.1038/s41370-024-00730-6

**Published:** 2024-11-09

**Authors:** Stephanie M. Holm, Brett C. Singer, Mi-Suk Kang Dufour, Woody Delp, James E. S. Nolan, P. Jacob Bueno de Mesquita, Bailey Ward, Yahna Williamson, O’Philia Le, Marion L. Russell, Kim G. Harley, John R. Balmes

**Affiliations:** 1 Division of Occupational, Environmental and Climate Medicine, Department of Medicine, University of California, San Francisco, San Francisco, CA USA; 2 Western States Pediatric Environmental Health Specialty Unit, San Francisco, CA 94143-0843 USA; 3 School of Public Health, University of California, Berkeley, Berkeley, CA USA; 4 Indoor Environment Group, Sustainable Energy and Environmental Systems Department, Lawrence Berkeley National Laboratory, Berkeley, CA USA; 5 Centre Hospitalier Universitaire Sainte-Justine, Montréal, QC Canada; 6 Department of Public Health, Roger Williams University, Bristol, RI USA

**Keywords:** Children, Cooking, Indoor air pollution, Video, Exhaust ventilation, Nitrogen dioxide

## Abstract

**Background:**

Cooking-related emissions contribute to air pollutants in the home and may influence children’s health outcomes.

**Objective:**

In this pilot study, we investigate the effects of a cooking ventilation intervention in homes with gas stoves, including a video-based educational intervention and range hood replacement (when needed) in children’s homes.

**Methods:**

This was a pilot (*n* = 14), before-after trial (clinicaltrials.gov #NCT04464720) in homes in the San Francisco Bay Area that had a school-aged child, a gas stove, and either a venting range hood or over-the-range microwave/hood. Cooking events, ventilation use, and indoor air pollution were measured in homes for 2–4 weeks, and children completed respiratory assessments. Midway, families received this intervention: (1) education about the hazards of cooking-related pollutants and benefits of both switching to back burners and using the range hood whenever cooking and (2) ensuring the range hood met airflow and sound performance standards. The educational intervention was delivered via a video developed in conjunction with local youth.

**Results:**

We found substantially increased use of back burners and slight increases in range hood use during cooking after intervening. Even though there was no change in cooking frequency or duration, these behavior changes resulted in decreases in nitrogen dioxide (NO2), including significant decreases in the total integrated concentration of NO2 over all cooking events from 1230 ppb*min (IQR 336, 7861) to 756 (IQR 84.0, 4210; *p* < 0.05) and NO2 collected on samplers over the entire pre- and post-intervention intervals from 10.4 ppb (IQR 3.5, 47.5) to 9.4 (IQR 3.0, 36.1; *p* < 0.005). There were smaller changes in PM2.5, and no changes were seen in respiratory outcomes.

**Impact:**

This pilot before-after trial evaluated the use of a four-minute educational video to improve cooking ventilation in homes with gas stoves and one or more school-aged children. Participant behavior changed after watching the video, and there were decreases in indoor air pollutant concentrations in the home, some of which were significant. This brief video is now publicly available in English and Spanish (wspehsu.ucsf.edu/projects/indoor-air-quality), and this provides suggestive evidence of the utility of this simple intervention, which could be particularly beneficial for households that have children with asthma.

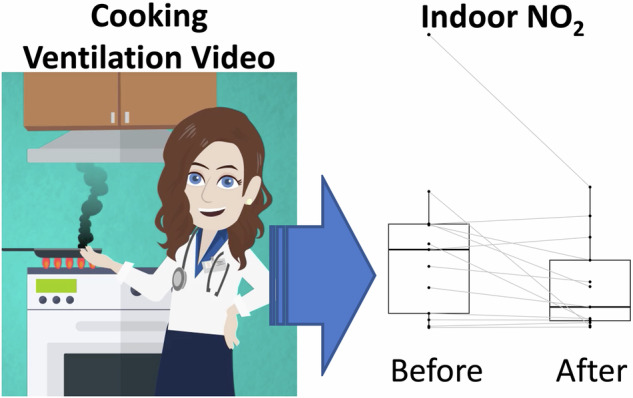

## Introduction

Indoor air pollution is increasingly recognized as an adverse contributor to human health as humans in high-income countries spend 80% or more of their time indoors [1, 2]. Indoor pollutants, including fine particulate matter (PM_2.5_) and nitrogen dioxide (NO_2_), are particularly problematic for individuals with underlying respiratory illnesses such as asthma, which affects one out of every 15 children in the US [3].

Cooking with any fuel and usage of gas stoves are both substantial contributors to indoor pollution through emissions of multiple air pollutants [4–11]. Some researchers have estimated that more than 7000–10,000 annual deaths in the US are attributable to pollution from cooking and heating in residences [12]. Because of air mixing throughout homes, prior studies report that increases in the gas concentrations in the kitchen are accompanied by increases throughout the living space [13]. A 2017 study in middle-class homes in Northern California found that 44% of homes exceeded the EPA outdoor 1-h NO_2_ air quality standard of 100 ppb during standardized cooking events performed by the investigators [5, 14, 15]. Homes with gas stoves can have NO_2_ concentrations much higher than those without them [14]. Because up to 65% of California households have gas stoves [16], more than 20 million people in the state may be regularly exposed to potentially hazardous concentrations of NO_2_. Gas cooking also produces PM_2.5_ [17], with cooking duration related to both PM_2.5_ and NO_2_ concentrations [6].

Both gas cooking and household NO_2_ concentrations are thought to contribute to asthma and childhood wheezing [18–20]. Studies of school-aged children have reported an association of gas cooking or cooking-related emissions with cough [21], daily NO_2_ exposure with nighttime inhaler use [22], and 72-h NO_2_ exposure with cough or nighttime symptoms [23]. Some studies have documented dose-dependent effects, with those from higher NO_2_ households reporting more frequent asthma symptoms [24]. An Institute of Medicine review in 2015 concluded that there is “sufficient evidence of an association” between brief high NO_2_ exposures and increased airway responsiveness in people with asthma [25]. Moreover, a large cross-sectional, nationally representative survey reported an association between parental reports of not ventilating gas stoves and higher odds of childhood cough [26], asthma [27], wheezing [27], and bronchitis [27].

Because there are emissions that result from gas cooking and evidence that these emissions may contribute to respiratory symptoms in children, strategies to mitigate those exposures are key. A recent study by our group reported that cooking exposures were an important contributor to indoor PM_2.5_ in a sample of urban households with asthmatic children; specifically, households that used range hoods for ventilation during cooking had lower PM_2.5_ concentrations [28]. An analysis of time-resolved PM_2.5_ measurements in 132 Canadian homes found that usage of kitchen ventilation (range hood use and/or window opening) resulted in faster removal of PM_2.5_ emitted during cooking [29]. In repeated, standardized cooking events, the use of a hood exhaust fan was found to decrease the PM_2.5_ concentrations, with the amount of decrease related to the fan flow rate [30]. The proportion of emissions captured by the exhaust hood (capture efficiency) is dependent on the dimensions of the hood, the flow rate [31], and the coverage of the burner by the hood, which is often much higher on back burners [32].

Many homes in North America have inadequate or no kitchen exhaust ventilation, including among buildings that have ostensibly installed building code-compliant devices [33]. Deficiencies include airflows that are significantly lower than required and high operating noise levels, which people report as an important factor limiting ventilation use [16]. Even when kitchen ventilation is adequate, it is inconsistently used [34, 35]; a recent, nationally representative survey from Canada showed that despite 90% of households having ventilation devices above their cooktops, most of which vented to the outdoors, only 30% of people reported regularly using it, most often during deep frying [35]. This is consistent with other work that demonstrates that people use the range hood more frequently during longer cooking events and during those events with higher PM_2.5_ emissions (e.g., frying), even though objectively measured use of range hoods tends to be substantially lower than self-reported use [34]. Importantly, respondents in the Canadian survey reported a willingness to increase their range hood use and to cook on the back burner more often once they learned about the potential pollution and health benefits [35].

Despite evidence that improved ventilation may improve respiratory symptoms–and that people are potentially willing to use cooking ventilation–interventions targeting cooking ventilation in the U.S. have been sparse. A randomized controlled trial of whole house ventilation (not specific to cooking times) among homes with children with asthma reported reduced volatile air pollutants (though no difference in NO_2_) and a decrease in wheezing episodes in the year after the increased ventilation [36]. The only study [37] that randomly assigned families to a cooking ventilation intervention saw no difference in NO_2_ concentrations following the installation of range hoods for 15 families in Baltimore, but that study did not assess whether the residents used the new range hood.

Environmental health interventions are an important component of preventive medicine and public health [38]. Even brief air pollution educational interventions have shown changes in reported behavior, both immediately after the intervention and for weeks afterward [39]. Best practices for the communication components of such interventions include tailoring the messaging to the community being served [40], getting direct feedback [41] from members of the community, and evaluating the effectiveness of such communications. Videos have been noted to be effective in creating behavior change [42, 43] and are a particularly cost-effective strategy, with the added benefit of reaching lower literacy individuals [43]. In this pilot study, we investigate the effects of a cooking ventilation intervention that included both range hood replacement and a video-based intervention in the homes of children.

## Materials and methods

### Study design

This was a pilot before-after trial in homes in the San Francisco Bay Area that had at least one school-aged child, a gas stove, and a venting range hood or over-the-range microwave and hood (OTR). The trial ran from July 2021 through June 2022 and assessed a cooking ventilation intervention consisting of (1) ensuring that the home had a working range hood that met both airflow and sound performance standards and (2) education about the hazards of cooking pollutants and the benefits of using the range hood whenever cooking occurs. The trial is registered with clinicaltrials.gov, #NCT04464720.

Because the funding for this study began (in March 2020) alongside the COVID-19 global pandemic, modifications to protocols were made to maintain as large a sample size as possible and advance the scientific goals while flexibly adapting to evolving university research guidelines, as well as the needs and comfort of study participants. These changes included (1) delaying the data collection start until the summer of 2021; (2) moving from a stepped wedge, randomized controlled trial to a before-after trial; (3) allowing some fluctuations in overall study duration and number of study visits; (4) having some study visits in the participants’ homes and others at an outdoor community site to minimize indoor contact time; and (5) expanding to including children regardless of asthma diagnosis.

Participant and study data were collected during a screening and follow-up phone call and 3-4 subsequent study visits. Written informed consent was obtained from all participants (as well as permission from the parent and assent of the child for their participation). During the first study visit, measurements of the range hood airflow and sound level were performed to assess if the existing range hood or OTR met the specified criteria (further explained in the ‘Intervention’ section below). Baseline data were collected from the air pollutant, stove, and range hood sensors (further delineated in the relevant sections below) that were installed during the initial visit through the intervention visit (either the second or third visit, depending on the protocol in place at the time of that participant’s enrollment). On average, the baseline lasted 13 days (range: 5–26 days).

At the intervention visit, all participants received a video-based educational intervention instructing the child and their guardian about cooking practices designed to decrease pollution exposure. Participants also received an associated infographic and magnet. If the range hood or OTR failed to meet the criteria at the initial visit, it was replaced either the same day or shortly before the intervention visit. The child performed spirometry and fractional exhaled nitric oxide (FeNO) measurements at the time of that intervention visit, reflecting their health status during the preceding baseline interval, and the parent completed questionnaires about both child health and cooking behaviors.

The post-intervention period then lasted from the intervention visit through the final visit (third or fourth visit overall; an average of 12 days, range 5–21 days). For each household, the post-intervention duration was usually similar to the duration of the pre-intervention study period, such that the intervention was delivered at roughly the midpoint of the entire study interval. The air pollution, stove, and range hood sensors remained in place for the entire data collection period–allowing for objective measurements of cooking intervals and range hood use both before and after the intervention–and were removed at the final visit. The child and parent completed the same questionnaires and health assessments as previously, as well as a close-out questionnaire.

### Study population

We recruited children ages 6–12 living in the East Bay area of California. They were eligible for this study if the parent reported that they had both a gas stove and a venting range hood, i.e., one that extracted air from the kitchen to the outdoors. They were excluded from the study if they lived with a smoker who smoked indoors, if they knew they would not have stable housing for the period of the study, or if they were not fluent in English. In cases where more than one child per household was eligible for enrollment, all were enrolled in the study, data were collected for all children, and data from the child with the most complete lung function data were selected for analyses (see ‘Health Outcome Assessments’ below). In total, 18 participants completed the study, representing 14 distinct households with four sibling pairs.

Recruitment primarily occurred through East Bay pediatric clinics via recruitment fliers and information cards. At a few clinics, postcard mailings were also sent to potentially eligible patients. We also advertised for the study on an institutional website, through social media, and via fliers at community spaces (libraries, community centers, etc.).

Information about the child, household, and participating parent was collected in the phone interview using the ISAAC Environmental Questionnaire and demographic questions.

### Intervention

To decrease the likelihood that inadequate ventilation equipment would mask the effects of the educational intervention, the existing range hood or OTR were assessed by a contractor for replacement if they did not meet the target criteria. These were (1) minimum airflow of 100 cubic feet per minute (cfm; the California building code requirement as of Nov. 2020) and (2) sound pressure (loudness) no greater than 60 A-weighted decibels (dBA, a sound level often associated with annoyance) at a distance of 2 m. When feasible, the participant (and building owner, as applicable) was offered a replacement hood or OTR that met the specifications.

All study participants received education regarding use of the range hood during all cooking events. The educational intervention consisted of a 4-minute animated video featuring one of the physician researchers. The video provided background about cooking-related pollution and encouraged participants to (1) always use their range hood, (2) use the back burners when cooking on the stovetop, and (3) move other cooking appliances closer to the range hood to use it during those cooking activities as well. Local youth study assistants provided crucial feedback during the development of the video to ensure that the information was presented in a way that resonated with the local community. Research assistants played the video for the parent and child via a portable tablet computer during the intervention study visit. Participants were given a printed infographic on cardstock, as well as a small magnet to post in the kitchen, with the takeaway points from the video (both from a still frame of the video). Weekly, the participants received text messages reminding them to continue using the tips from the video. Adaptations of these educational materials have since been made publicly available at: https://wspehsu.ucsf.edu/projects/indoor-air-quality/.

### Air pollution assessments

Concentrations of time-resolved PM_2.5_, NO_2_, CO_2_, and other parameters were measured in the primary living area of each home (to represent the exposure received by the occupants) and typically logged every 1 min throughout the study interval. The air contaminants were measured along with temperature and relative humidity using an eLichens Indoor Air Quality Pro Monitor (eLichens, Grenoble, France). The eLichens has no integrated display and household members did not receive any feedback from the device other than an indicator LED to show that the device was operating. Integrated NO_X_ and NO_2_ samples were collected during the intervals between study visits using Ogawa Passive Samplers (Ogawa USA [44], Pompano, Florida), placed in duplicate adjacent to the eLichens real-time monitor.

### Cooking and kitchen exhaust ventilation monitoring

At the initial study visit, the airflow of each range hood or over-the-range microwave exhaust fan (OTR) present in the home was measured by a home performance contractor with expertise in ventilation equipment diagnostics to assess the need for intervention. Airflow was measured using a balanced-pressure flow hood method described by Walker and Wray [14] and used in several [45, 46] recent residential IAQ field studies. The measurement was made starting at the lowest fan speed setting and progressing to higher speed settings to identify the minimum setting needed to move 100 cfm. At that setting, a measurement was also made of the A-weighted sound pressure decibels (dB-A) at a 2-meter distance using a smartphone with an SPL Audio Tools app.

For the duration of the study interval, stove, oven, and range hood/OTR use were measured at 1-minute intervals using Lascar Easylog Thermocouple Data Loggers (with one thermocouple placed adjacent to each of the four cooktop burners), Onset Hobo temperature loggers above the cooktop (affixed to the range hood), and a Digisense Data Logging Vane Anemometer, affixed to the air inlet of the range hood. We used these temperature data to derive cooking intervals and the logged real-time airflow velocity data to monitor the frequency of ventilation use. Self-reported cooking data were also collected at each visit.

### Health outcome assessments

Baseline health information included an assessment of pre-existing medical conditions, including asthma (using the ISAAC Asthma questionnaire). Health outcomes included objective measures of respiratory health in all children at follow-up study visits.

With an EasyOne Spirometer, each child performed spirometry to complete three acceptable efforts (maximum eight attempts) in accordance with standard ATS/ERS performance criteria. Each effort was graded by two trained physicians in accordance with the 2019 ATS/ERS guidelines. If there were two or more efforts with acceptable quality and reproducible FEV_1_ and FVC (each ≤0.15 L), the best FEV_1_ and best FVC were used. If two or more efforts had usable quality and reproducible FEV_1_ but unacceptable FVC, only the best FEV_1_ was used, and FVC was considered missing.

Prior to spirometry, each child also had FeNO measured in accordance with ATS/ERS criteria by blowing a steady, sustained exhalation into a NIOX Vero device to complete two measurements with exhalation duration greater than 10 s and within 10% of each other (maximum eight attempts). If there were two measurements within 10%, this testing session was graded as acceptable, and the larger value was used. If there were one or multiple FeNO measurements of adequate duration (but none within 10%), this testing session was graded as usable, and these values were averaged to create the FeNO value for analysis. Children with asthma also completed the self-reported asthma control test with their guardian at each follow-up visit.

Because performing lung function maneuvers requires coordination of breathing in an unfamiliar manner, missing data for lung function test results is not necessarily related to underlying respiratory status and is common, especially in studies assessing children. Thus, lung function testing was performed in all interested children within the household and the child with the least missingness in the respiratory data was used as the primary study participant.

### Laboratory analyses

Ogawa samplers were hand-delivered from field to lab staff and kept cool on ice during transport. Twenty primary samplers were deployed, with 15 duplicates and five travel blanks. All Ogawa samples were extracted for analysis within 30 days from when the samplers were assembled. The samples were extracted and analyzed following the protocols provided by the company and as performed previously by our group (Ogawa USA, 2017) [44, 45, 47]. We computed mean temperature (T) and relative humidity (RH) for each Ogawa passive sampler deployment period based on measurements of the co-located eLichens monitors; as per the protocol, these values were then used to calculate the ambient NO_2_ concentrations from the measurement of the filter mass.

### Statistical analyses

Data analyses were performed using Python and R in accordance with a prespecified data analysis plan posted to Open Science Framework (https://osf.io/vbm9g/?view_only=fe4ae01f9100486c9f78e93804b14583). Briefly, air pollution sensors and thermocouples were cross-calibrated before deployment, and all study data had an initial quality assurance, visual review, and alignment of timestamps for equipment deployed together. Data processing included algorithmic identification of burner use events from thermocouple data, cooking events (burner events within 30 min of each other), range hood use events from anemometer data, and pollutant events (with the associated time and pollutant statistics) from the eLichens data.

For each cooking event, we calculated several metrics, including: the duration of use for each burner (called burner-time), the number and position of burners used (front and/or back, if it could be clearly determined), the cooking event duration (from the start of the first burner event until the end of the last burner event, among burner events separated by less than 30 min), whether there were anemometer readings to indicate range hood use during the event, the number of minutes the anemometer recorded airflow through the hood, whether there were valid data for pollutants in that same time interval (PM_2.5_ in µg/m^3^; NO_2_ in ppb; CO_2_ in ppm), the maximal 10-min average concentration above the baseline for each pollutant during the cooking event (called the event peak), the event integrated concentration of the pollutant above the background level (concentration*min) during the cooking event, and a normalized event integrated concentration by dividing by the duration of the event (in burner-time).

Data were summarized within each household and study interval, which included the number of cooking events per day and their duration, as well as the number and percent of cooking events with range hood use. To capture episodes with intentional range hood use for most of the cooking event, we also determined the number and percent of events with the range hood on for ≥80% of the cooking time, as has been done previously [34]. For the pollutants, we calculated not only the means across the cooking events within each household and interval for each of the metrics calculated for the cooking events, but also the total integrated concentration (the sum of the integrated concentration across cooking events in that interval). This approximates the contribution of cooking to this pollutant in the house over the measured time interval.

Wilcoxon Signed Rank tests were used to compare the paired pre-intervention to the post-intervention data to assess the relationship between intervention status and the following groups of exposure variables: range hood use, burner use, and pollutant concentrations during cooking events (PM_2.5_, NO_2_ and CO_2_). The pre-specified alpha value was 0.05, so values of p below 0.05 were considered significant. Wilcoxon Signed Rank tests were also used to compare the pre-intervention data to the post-intervention data to assess the relationship between intervention status and the following health outcome variables: airways inflammation (i.e., FeNO) and lung function. Further details of the exact variables calculated are available in the supplemental material. Due to the small final sample size, the study was underpowered to assess health differences, no imputation was performed, data were analyzed in a complete case fashion, and corrections for multiple comparisons were not feasible. Median values reported in the results and tables/figures are the median values of the mean within each household for each time period.

## Results

Among the 14 households and 18 children that participated in the study, a total of 14 children and their unique households (Table 1) are included in the final study sample, by selecting for the child in the home with more complete health data (Supplementary Fig. S1 illustrates participant flow through the screening process). Possibly related to the overburdening of families of lower socioeconomic status (SES) during the COVID-19 pandemic, the final sample was of fairly high SES. Among children who enrolled in the study, there were more who were non-Latinx white, and who had allergies and/or eczema, compared to the complete set of children screened (Supplementary Table S1).

**Table 1:** Child participant and household characteristics (*n* = 14).

Child participant characteristics	
	Number (percent) or Mean (SD)
Gender	
Girl	7 (50.0%)
Boy	7 (50.0%)
Other gender identities	0 (0.0%)
Sex	
Female	7 (50.0%)
Male	7 (50.0%)
Race/Ethnicity	
Latinx	4 (28.6%)
Non-Latinx white	8 (57.1%)
Other	2 (14.3%)
Age	8.7 (2.0)
BMI	16.1 (2.6)
Weekly vigorous physical activity	
Never or occasionally	1 (7.1%)
Once or twice per week	2 (14.3%)
Three or more times a week	11 (78.6%)
History of Asthma (Yes)	4 (28.6%)
History of Allergies (Yes)	6 (42.9%)
History of Eczema (Yes)	6 (42.9%)
History of Prematurity (Yes)	1 (7.1%)

Household characteristics	
Household members	4 (0)
Housing type	
2–4 unit condo or apartment	1 (7.1%)
Single family detached	11 (78.6%)
Townhome or duplex	2 (14.3%)
Type of kitchen ventilation	
Combo with a microwave	2 (14.3%)
Standalone range hood	12 (85.7%)
Smoker living in the home (Yes)	0 (0.0%)
Household has other gas appliances (Yes)	12 (85.7%)
Highest educational level completed by the parent	
Graduate or professional degree	11 (78.6%)
Graduated high school or GED	1 (7.1%)
Some college	2 (14.3%)

At the time of the baseline measurements, the median measured flow through participant range hoods on the lowest setting was 159 cfm, with three (21%) households identified for replacement for not meeting the flow criterion of > 100 cfm (Supplementary Table S2, Supplementary Fig. S2). The median measured sound, 2 meters away from the range hood, was 56 dbA, with five (36%) households identified for a replacement for not meeting the sound criterion of <60 dB (Supplementary Table S2, Supplementary Fig. S3). In total, eight households were identified for potential range hood replacement with six of these receiving successful range hood replacement and/or minor ductwork prior to the end of the data collection interval. Reasons for unsuccessful replacements included difficulties in managing family and contractor schedules (made more difficult by pandemic-related factors), no available range hood in the needed size, and participant refusal to replace. All households that had range hood replacements met the sound criterion of <50 dB following the replacement, though not all met the flow criterion (Supplementary Figs. 2 and 3), likely due to airflow restrictions in the exhaust ducting.

**Fig. 1:**
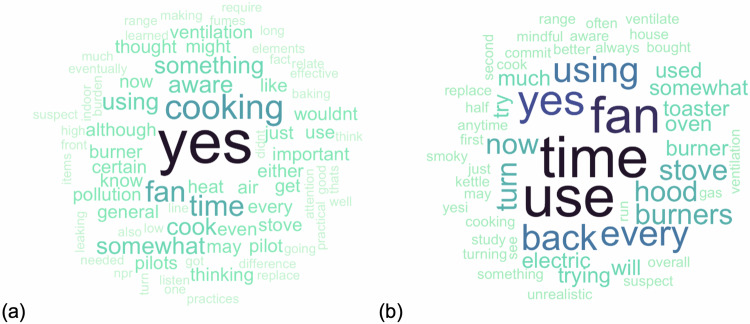
Words used by participants to describe changes after the intervention. These word clouds depict a qualitative summary of the free-text participant responses to questions about whether their (**a**) thinking and (**b**) behavior had changed regarding ventilation. Larger and more central words are those mentioned most frequently. In the (**a**) word cloud responding to whether their thoughts had changed, the most commonly used word by far was “yes”, with other commonly used words including topical words such as ‘cooking’, ‘fan’, ‘time’, ‘aware’ and ‘pollution’. In the (**b**) word cloud responding to whether their behavior had changed, the responses are less definitive (yes is no longer the most common word), with words indicating uncertainty showing up more prominently (e.g. “try”, “suspect”, “trying” and “unrealistic”). References to increased time using various interventions were common with mentions of both back burners and range hoods.

Of those who provided feedback on the educational video, 11/11 (100%) reported that it was understandable; nine (82%) reported trusting the information in the video “very much,” and two (18%) trusted the information “somewhat”; four (36%) said they found the video very visually pleasing and seven (64%) said it was somewhat visually pleasing. Generally positive feedback was also reported on the study experience overall. When asked whether their experience with the study changed how they thought about cooking ventilation and how they behaved regarding cooking ventilation, participants stated much more strongly that their thinking had been changed (Fig. 1a), but most still reported that there were some behavioral changes they had made and anticipated continuing (Fig. 1b). Example statements from participants included: “Yes--I used to only use the fan if cooking something smoky and now am trying to use all the time,” and “I may use the back burner somewhat more, and may use the fan somewhat more, but it’s unrealistic to commit to both all of the time. I suspect our overall use of both will be more than it was before, but not as much as during the second half of the study.”

All 14 households had both pre- and post-intervention cooking event data (from temperature monitoring, Table 2 and Supplementary Fig. S4). Due to instrument or technician errors, some air pollutant and range hood use data were missing. One household had no recorded real-time pollution data (pre or post), one household was missing post-intervention averaged NO_2_ data (from the passive samplers), one had no pre-intervention range hood data, and one had no post-intervention range hood data.

**Table 2:** Characteristics of the measured cooking events during the pre- and post-intervention intervals, summarized among all 14 households.

Cooking event characteristics		
	Pre-Intervention Period Median of household means (range)	Post-Intervention Period Median of household means (range)
Number of cooking events per day	2.6 (0.6, 5.2)	2.7 (0.6, 3.8)
Burners used per cooking event	1.3 (1.0, 1.7)	1.3 (1.0, 1.8)
Duration of cooking events (min)	35.2 (25.0, 61.0)	38.4 (25.6, 61.6)
Burner-time per day (min)	107.2 (17.6, 166.8)	106.3 (18.7, 204.3)
Front burner-time per day (min)	84.0 (7.6, 156.7)	45.8 (7.3, 163.4
% of burner-time that used a front burner	78% (13, 94)	43% (10, 92)
Back burner-time per day (min)	7.8 (0.0, 95.6)	41.5 (0.0, 180.5)
% of burner-time that used a back burner	6% (0, 71)	38% (0, 88)
Percentage of cooking events for which there were valid range hood flow data	100 (0, 100)	100.0 (0, 100)
Delay from start of cooking event to start of range hood use (min)	1.6 (0.0, 8.2)	1.6 (0.0, 8.1)
Number of cooking events with range hood use per day (n)	0.5 (0.0, 3.4)	0.8 (0.0, 2.7)
Percent of cooking events with any range hood use	23.8 (0.0, 100.0)	54.2 (0.0, 100.0)
Percent of cooking events with range hood use for ≥80% of the event	3.3 (0.0, 66.7)	3.7 (0.0, 77.8)

**Fig. 2:**
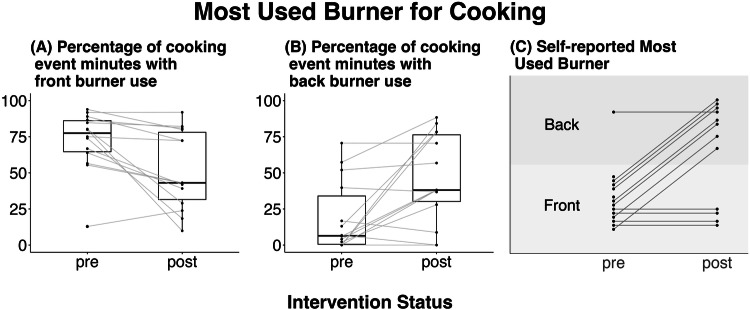
Changes in burner use behavior following the intervention. **A** Percentage of cooking time (min) within households that included the use of a front burner (*p* < 0.005). **B** Percentage of cooking time (min) within households that included the use of a back burner (*p* < 0.05). **C** Participants’ self-reported most commonly used burner over the prior week (*p* < 0.05). All p values are from Wilcoxon signed rank tests.

Following the intervention, the duration of cooking was unchanged with burner-time per day going from 107.2 (17.6, 166.8) to 106.3 (18.7, 204.3) minutes (Table 2). Burner use shifted substantially from front to back burners, both as reported by the study participants and as measured with objective temperature data (Fig. 2, Supplementary Table S3). The percentage of burner-time that included the use of a front burner decreased from a median of 78% (range 13–94) during the pre-intervention period to 43% (range 10–92, *p* < 0.005) following the intervention. Correspondingly, the percent of burner-time that included the use of a back burner increased from 6% (range 0–7) to 38% (range 0–88, *p* < 0.05). Participants’ perception of their behavior change exceeded the measured change: in the pre-intervention period 9/14 (64%) reported a front burner as their primary burner, while only 4/12 (33%, *p* < 0.05) reported still using a front burner as their primary burner following the intervention. However, the participants’ reports did generally reflect whether there had been a major shift in their use or not; those who reported no change in front to back burner use had a median decrease of 0.1% in their back burner use, whereas those who reported changing their primary burner from front to back had a median increase in back burner use of 32%.

**Fig. 3:**
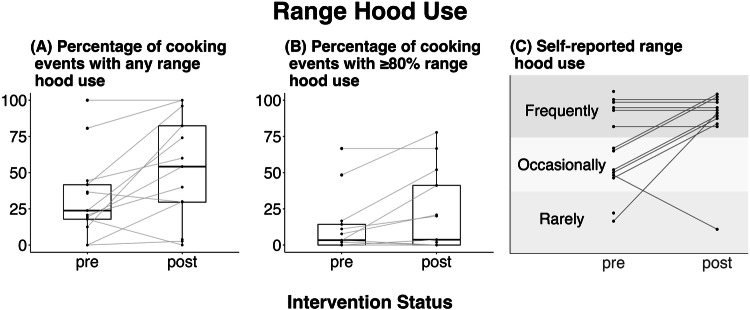
Changes in range hood use behavior following the intervention. **A** Percentage of cooking events within each household that included any measured range hood use (*p* < 0.05). **B** Percentage of cooking events that included measured range hood use for 80% of the cooking event time or more (*p* < 0.005). **C** Participants’ self-reported frequency of range hood use (*p* = 0.12). All p values are from Wilcoxon signed rank tests.

While self-reported range-hood use did not increase in a statistically significant way following the interventions, vane anemometer measurements showed that many households did substantially increase their range-hood use (Fig. 3). Participants did report an increase (though non-significant) in their own range hood use (*p* = 0.12). The median percent of cooking events where range hoods were used at all was 24% in the pre-intervention phase and 54% post-intervention (*p* < 0.05). The median percent of cooking events with range hood use for 80% or more of the cooking event time remained low in both pre-and post-intervention phases (3 and 4%, respectively, *p* < 0.05), even though it demonstrated a statistically significant increase. Range hood use was similar when restricted to cooking events where pollution was detected (an increase in NO_2_ and/or PM_2.5_; Supplementary Table S2, Supplementary Figs. S5 and S6), and there was no change seen in the time it took participants to turn the range hood on after beginning to cook (Supplementary Fig. S7). There was also no clear temporal trend in range hood use over the (short) follow-up duration, or in relation to text reminders sent to the family.

**Fig. 4:**
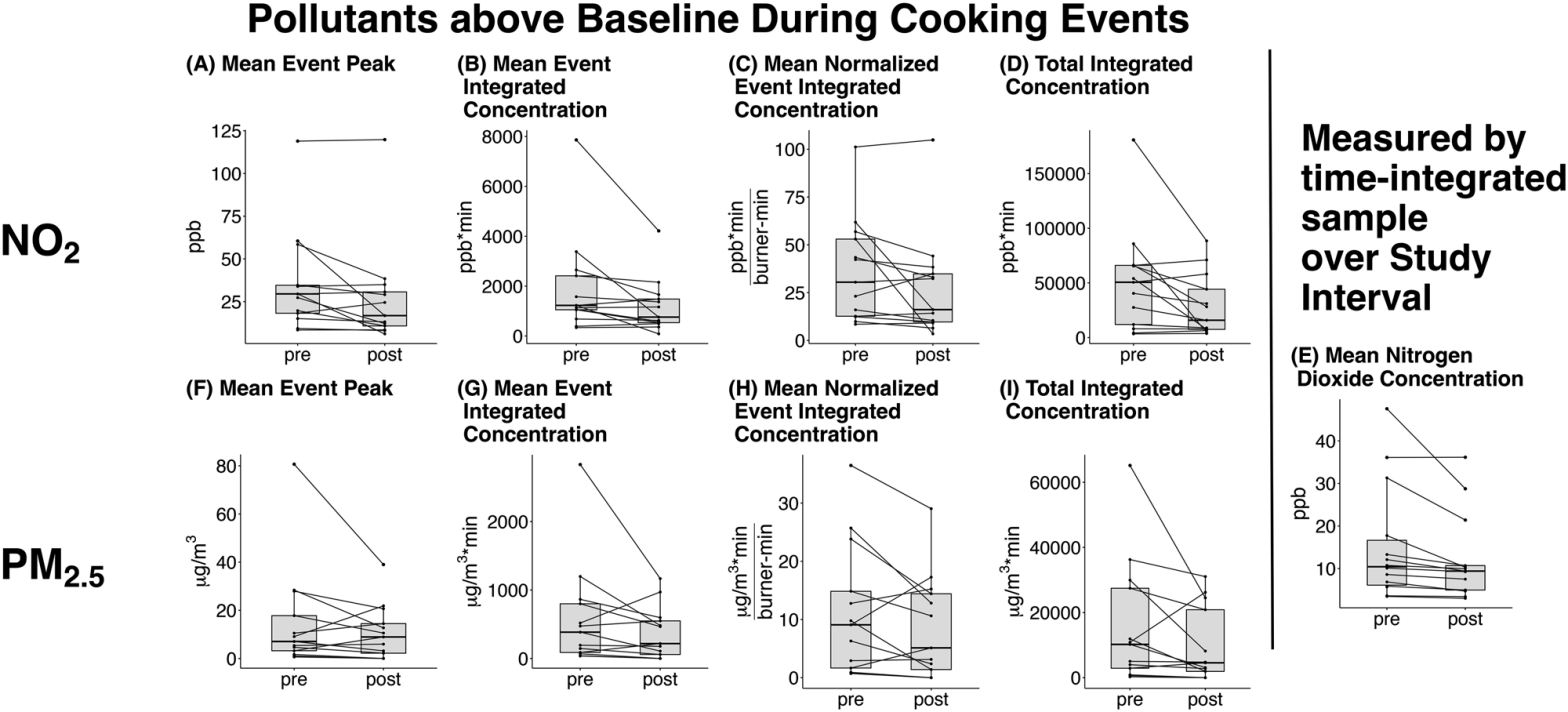
Changes in measured pollutant concentrations following the intervention. **A**–**E** Changes in NO2 concentrations, **F**–**I** Changes in PM2.5 concentrations. **A** and **F** illustrate changes in the mean event peak during cooking events for NO2 (ppb, panel **A**, *p* = 0.059) and for PM2.5 (µg/m3, panel **F**, *p* = 0.21); **B** and **G** illustrate changes in the mean event integrated concentration during cooking events for NO2 (ppb*min, panel **B**, *p* < 0.05) and for PM2.5 (µg/m3*min, panel **G**, *p* = 0.12); **C** and **H** illustrate changes in the mean event integrated concentration normalized to time for NO2 ((ppb*min)/burner-min, panel **C**, *p* = 0.11) and for PM2.5 ((µg/m3*min)/burner-min, panel **H**, *p* = 0.11); **D** and **I** illustrate changes in the total integrated concentration during cooking events for NO2 (ppb*min, panel **D**, *p* < 0.05) and for PM2.5 (µg/m3*min, panel **I**, *p* = 0.05); and **E** illustrates the mean NO2 concentration time-integrated over the entire observation window (not only cooking events) and measured on a passive sampler (ppb, *p* < 0.005). All p values are from Wilcoxon signed rank tests.

Consistent with the shift in front to back burner use and the potential increase in range hood use, there were substantial differences in pollutant concentrations within the homes in the post-intervention period compared to the pre-intervention (Table 3 has definitions of the various pollutant metrics, and Fig. 4 displays these results), even though cooking duration did not change. The mean NO_2_ event peak (or highest 10-min average over baseline) occurring during cooking events decreased 12.7 ppb following the intervention (from 29.5 (IQR 8.4, 119) to 16.8 (IQR 6.1,120), *p* = 0.06), and the mean event integrated NO_2_ concentration decreased 470 ppb*min per cooking event (from 1230 (IQR 336, 7860) to 756 (IQR 84.0, 4210), *p* < 0.05). The mean normalized event integrated concentration ((ppb*min)/burner-min) decreased 14.3 from 30.4 (IQR 8.4, 101) to 16.1 (IQR 3.4, 105), *p* = 0.11). Importantly for human health, the total integrated NO_2_ concentration decreased 34,800 ppb*min totaled over all cooking events in the pre and post intervals (from 50,600 (IQR 3400, 180,000) to 15,900 (IQR 3740, 88,500), *p* < 0.05). Averaged NO_2_ concentrations_,_ as measured on time-integrated passive samples for the entire pre/post interval durations, also decreased by 1.0 ppb (from 10.4 (IQR 3.5, 47.5) to 9.4 (IQR 3.0, 36.1), *p* < 0.005). Though some households also had substantial decreases in PM_2.5_ concentrations, these were less impressive (Fig. 4, Supplementary Table S4). However, the substantial and significant decrease in total integrated PM_2.5_ concentration across all cooking events (from 10,200 (IQR 275, 65,200) to 4570 (IQR 0.0, 31,000), *p* = 0.05) suggests that a possible effect may be obscured by small sample size and the inherent variability among individual events. There were also small, non-significant decreases in event peak CO_2_ of 70.9 ppm (from 985 (IQR 679, 2060) to 914 (IQR 560, 2330), *p* = 0.18, Supplementary Fig. S8).

**Table 3:** Definition of air pollution metrics calculated for each household from time-resolved sensor data.

Name	Calculation	Relevance
Baseline	*Time-varying value using a robust routine based on a LOESS technique over an 8-hour rolling window. The primary contributions to this are continuous indoor sources and pollutants entering from the outdoors, which vary slowly*.	This provides an estimate of contributions from all other sources.
Event Peak	*Highest 10-minute average concentration – baseline*	This is a measure of the maximum increase caused by the cooking event.
Event Integrated Concentration	$\int ({concentration}-{baseline})$	This is a measure of the total pollutant contribution from a single cooking event.
Normalized Event Integrated Concentration	$\frac{\int ({concentration}-{baseline})}{{burner\; minutes}}$	This is the pollutant contribution from a single cooking event, normalized to the total burner use.
Total Integrated Concentration	${\sum}_{{interval}({pre\; or\; post})}\int ({concentration}-{baseline})$	This is a measure of the total pollutant contribution from all the cooking events that occurred in a house within an interval (either pre or post).

These were calculated in accordance with a pre-specified analysis plan posted to Open Science Framework at https://osf.io/vbm9g/?view_only=fe4ae01f9100486c9f78e93804b14583.

There was a marginal worsening in lung inflammation and a larger increase in lung function following the intervention (Supplementary Table S5, Supplementary Figs. S9–S14). Median FeNO increased 2.8 ppb from the measurement just prior to the intervention (*p* = 0.058), and FEV_1_ decreased one z score (*p* = 0.056). Despite these nearly significant changes in FEV_1_ and FeNO, the FEV_1_/FVC ratio was unchanged (*p* = 0.89), suggesting that the children’s overall ability to execute the test maneuvers may have played a large role. The measurement-to-measurement variability at different testing points during the study was quite large, which could be consistent with variable results due to learning of the maneuvers. As planned, we created exploratory plots to look at changes in respiratory status related to the pollutants that had been found to change. Thus, FEV_1_ and FeNO were each plotted against the total cooking event integrated PM_2.5_ and NO_2_, concentrations and the total study interval integrated NO_2_ concentration. No clear patterns emerged (Supplementary Figs. S15–S20).

Prespecified sensitivity analyses demonstrated (Supplementary Table S6) no real differences in behavior or pollutant concentrations when excluding the participants who had two visits prior to intervention compared to one (*n* = 12). When eliminating the households that did not receive indicated range hood replacements, the only difference among the behavior and pollutant analyses was that participants were more likely to self-report more range hood use (*p* = 0.07; *n* = 11). When restricting the cohort to atopic participants (those with allergic rhinitis, eczema, and/or asthma, *n* = 9), all findings became less significant, likely due to the smaller sample size. Notably, among all sensitivity analyses, none of the respiratory tests showed significant differences and were clearly less significant than in the main model.

## Discussion

In this small, before-after pilot study, we observed changes in participant behavior related to range hood use and cooking burner selection after a brief video-based educational intervention. Objectively measured cooking and ventilation data demonstrated that–despite similar cooking patterns (frequency and duration) before and after the intervention–the participants cooked more often on the back burner and increased range hood use post-intervention. Consistent with these behavioral changes, there were changes in measured NO_2_ and some changes in PM_2.5_ concentrations during cooking events.

We did not find any differences in respiratory status among the children; this is likely in part because more of the children in our sample did not have asthma and generally had healthy and stable airways. In addition, though the pollutant and behavior data were measured continuously over time and then aggregated to pre and post-intervals, the respiratory outcome data are individual point-in-time snapshots and thus more susceptible to variability. Because this was a pilot study, changes in other factors (such as outdoor air pollution, allergen presence, or infectious disease presence) may have introduced sufficient variability to overwhelm any cooking-ventilation-related changes in our small sample.

The intervention developed in this study was based on a health belief model [48] framework, where factors influencing health-related behaviors include perceived risks and benefits, barriers, and self-efficacy. The 4-minute video covered the risks and benefits of the intervention, using simple language and repetition to improve participant self-efficacy. Community feedback also helped guide the video development, particularly the visuals. Because perceived noisiness is a common complaint and barrier to using range hoods [35], we aimed to minimize that barrier by replacing hoods that were found to be too noisy. We also applied elements of social cognition theory [43] by focusing on two low barrier-interventions (ventilating during cooking, specifically with a range hood when available; and cooking on back burners to improve the range hood effectiveness) and modeling the health-protective behavior by showing the character in the video improving her range hood use. The intervention video is now publicly available online in English and Spanish (https://wspehsu.ucsf.edu/projects/indoor-air-quality/) and has been integrated into at least one large asthma triggers home mitigation program. We know of no other cooking ventilation education interventions targeted at homes with children that have had rigorous evaluations.

Mechanistic studies of PM-related health effects point to multiple modes of action in the body, including the induction of oxidative stress and inflammation [49]. These mechanisms can directly increase respiratory symptoms and decrease lung function. Histological changes seen with NO_2_ exposure include eosinophilic and neutrophilic inflammation [50], which can contribute to asthma symptoms. Human exposure studies have also reported that exposure to NO_2_ amplifies the effect of allergens on the lung function of allergic asthmatics [51]. We did not see changes in measured respiratory outcomes, likely partially related to the short duration of follow-up after the intervention in this pilot study. Because this study focused on air pollution measured at the household level, there is also inherent misclassification when this is used as a proxy for children’s exposure, and concentration reductions assessed in the main living area in the home may be larger than the children’s exposure reduction. Despite this, because of the mechanisms by which these pollutants are known to affect respiratory health, decreasing indoor pollutant concentrations could have substantial health improvements for children and their households, even if personal exposure differences are smaller than those measured in the main living areas.

Multiple characteristics of our specific study population could affect the size of the measured relationship between the educational intervention and the pollutant concentrations in the homes. Due to recruitment limitations related to the COVID-19 pandemic, the final study population was of high socioeconomic status, more likely to be white than the local population and mostly living in single family homes. Prior work demonstrates that cooking-related NO_2_ concentrations are higher in smaller dwellings like apartments compared to larger single family homes [33], suggesting that the indoor air quality improvements associated with cooking ventilation may be most substantial in smaller dwellings. Thus, we may have underestimated the true potential benefit of the intervention. There can also be large differences in the production of cooking-related pollutants based on what is being cooked and how [52]; different cooking oils, for example, produce wildly different pollutant concentrations during typical cooking conditions because of differences in their smoke points [53]. Detailed assessments of the cooking behaviors and cuisine selection of our participants was outside of the scope for this small study, but the more homogenous socioeconomic, racial and ethnic makeup of the study population suggests that their cooking and cuisine preferences likely do not reflect the general population, even in their local area. This could affect the pollutant production, and therefore the estimated benefit from the educational intervention, in ways that would be difficult to predict.

Our sensitivity analyses suggest that the findings are not driven by small subsets of our sample. The number of contact points with our investigators did not seem to substantially affect behavior changes, and the behavior didn’t shift over the period of the study. Though participants that got a new range hood were more aware of increasing their range hood use, measured differences were no different when including those that did not get a new range hood.

Finally, it has been well-described that behavior changes that occur in the setting of research studies may be less likely to occur and persist in real-world settings, especially over long periods of time. Having visible sensors in their home and recent discussions of cooking and ventilation may heighten perceived risk and may have influenced participant behavior even prior to our intervention. While we attempted to learn about this through questionnaires, it is impossible to know the full picture. Regardless, our intervention was able to produce improvements in household air pollution that increased from the period when the sensors were first placed. Our intervention included not only the brief educational video, but multiple modalities to remind the participants of what had been learned in the video. Though an adapted version of the infographic is freely available and could be printed as either a card or magnet as we did in the study, not all viewers would do so. Nor would they have a third party reminding them of what they had learned. This may have created a setting in which behavior change was more likely in our study than it would be outside it. However, because this is an intervention that can easily be adopted by multi-modal asthma home visiting programs, many participants could encounter this intervention in settings not dissimilar from our study.

Thus, while some features of our study may overestimate the effect of the intervention and some could underestimate the effect, the potential to decrease indoor air pollution exposure in children’s homes with such a simple and brief educational intervention is promising. Though not ubiquitous, many homes have functioning range hoods that largely sit unused, making improved range hood use a low-barrier and potentially high-yield behavioral change, especially for households who have one or more members with respiratory disease. By evaluating the effectiveness of this intervention in a small number of homes with children, we were able to demonstrate short-term significant changes in indoor pollutant concentrations, though the small sample size limited our ability to randomize the intervention or adequately assess health outcomes. Future work planned in our group will leverage this video intervention in combination with cooking electrification interventions (that eliminate NO_2_ production) to assess their use in combination, with a larger sample size of children with asthma, and following the families for a longer duration.

## Supplementary information

Supplemental information

Reproducibility and quality checklist AIM

## Data Availability

Data and scripts to reproduce analyses are available from the authors upon request.

## References

[1] Klepeis NE, Nelson WC, Ott WR, Robinson JP, Tsang AM, Switzer P, et al. The National Human Activity Pattern Survey (NHAPS): a resource for assessing exposure to environmental pollutants. J Expo Sci Environ Epidemiol. 2001;11:231–52. 10.1038/sj.jea.7500165 11477521

[2] Leech JA, Nelson WC, Burnett RT, Aaron S, Raizenne ME. It’s about time: A comparison of Canadian and American time–activity patterns. J Expo Sci Environ Epidemiol. 2002;12:427–32. 10.1038/sj.jea.7500244 12415491

[3] Centers for Disease Control and Prevention. Most Recent National Asthma Data | CDC. 2023.https://www.cdc.gov/asthma/most_recent_national_asthma_data.htm (accessed 4 Feb2024).

[4] Habre R, Coull B, Moshier E, Godbold J, Grunin A, Nath A, et al. Sources of indoor air pollution in New York City residences of asthmatic children. J Expo Sci Environ Epidemiol. 2014;24:269–78. 24169876 10.1038/jes.2013.74

[5] Singer BC, Pass RZ, Delp WW, Lorenzetti DM, Maddalena RL. Pollutant concentrations and emission rates from natural gas cooking burners without and with range hood exhaust in nine California homes. Build Environ. 2017;122:215–29.

[6] Baxter LK, Clougherty JE, Laden F, Levy JI. Predictors of concentrations of nitrogen dioxide, fine particulate matter, and particle constituents inside of lower socioeconomic status urban homes. J Expo Sci Environ Epidemiol. 2007;17:433–44. 17051138 10.1038/sj.jes.7500532

[7] Heinrich J. Influence of indoor factors in dwellings on the development of childhood asthma. Int J Hyg Environ Health. 2011;214:1–25. 20851050 10.1016/j.ijheh.2010.08.009

[8] Kashtan YS, Nicholson M, Finnegan C, Ouyang Z, Lebel ED, Michanowicz DR, et al. Gas and Propane Combustion from Stoves Emits Benzene and Increases Indoor Air Pollution. Environ Sci Technol. 2023;57:9653–63. 37319002 10.1021/acs.est.2c09289 PMC10324305

[9] Kashtan Y, Nicholson M, Finnegan CJ, Ouyang Z, Garg A, Lebel ED, et al. Nitrogen dioxide exposure, health outcomes, and associated demographic disparities due to gas and propane combustion by U.S. stoves. Sci Adv. 2024;10:eadm8680. 38701214 10.1126/sciadv.adm8680 PMC11068006

[10] Fortmann, R, et al. Indoor Air Quality: Residential Cooking Exposures. Prepared for the California Air Resources Board, 2001.

[11] Dennekamp M, Howarth S, Dick C, Cherrie J, Donaldson K, Seaton A. Ultrafine particles and nitrogen oxides generated by gas and electric cooking. Occup Environ Med. 2001;58:511–6. 11452045 10.1136/oem.58.8.511 PMC1740176

[12] Thakrar SK, Balasubramanian S, Adams PJ, Azevedo IML, Muller NZ, Pandis SN, et al. Reducing Mortality from Air Pollution in the United States by Targeting Specific Emission Sources. Environ Sci Technol Lett. 2020;7:639–45.

[13] Singer BC, Delp WW, Lorenzetti DM, Maddalena RL Pollutant Concentrations and Emission Rates from Scripted Natural Gas Cooking Burner Use in Nine Northern California Homes. 2016.

[14] Mullen NA, Li J, Russell ML, Spears M, Less BD, Singer BC. Results of the California Healthy Homes Indoor Air Quality Study of 2011-3: impact of natural gas appliances on air pollutant concentrations. Indoor Air. 2016;26:231–45. 25647016 10.1111/ina.12190

[15] Logue JM, Klepeis NE, Lobscheid AB, Singer BC. Pollutant Exposures from Natural Gas Cooking Burners: A Simulation-Based Assessment for Southern California. Environ Health Perspect. 2014;122:43–50. 24192135 10.1289/ehp.1306673 PMC3888569

[16] Klug V, Lobscheid A, Singer B Cooking Appliance Use in California Homes – Data Collected from a Web-Based Survey. Lawrence Berkeley National Laboratory: Berkeley, CA, 2011.

[17] Hu T, Singer BC, Logue JM Compilation of Published PM2.5 Emission Rates for Cooking, Candles and Incense for Use in Modeling of Exposures in Residences. 2012.

[18] Lin W, Brunekreef B, Gehring U. Meta-analysis of the effects of indoor nitrogen dioxide and gas cooking on asthma and wheeze in children. Int J Epidemiol. 2013;42:1724–37. 23962958 10.1093/ije/dyt150

[19] Vieira SE, Stein RT, Ferraro AA, Pastro LD, Pedro SSC, Lemos M *et al*. Urban Air Pollutants Are Significant Risk Factors for Asthma and Pneumonia in Children: The Influence of Location on the Measurement of Pollutants. *Arch Bronconeumol*: 7. 10.1016/j.arbres.2012.05.005 22763046

[20] Sharma HP, Hansel NN, Matsui E, Diette GB, Eggleston P, Breysse P. Indoor Environmental Influences on Children’s Asthma. Pediatr Clin North Am. 2007;54:103–20. 17306686 10.1016/j.pcl.2006.11.007

[21] Behrens T, Maziak W, Weiland SK, Rzehak P, Siebert E, Keil U. Symptoms of Asthma and the Home Environment. The ISAAC I and III Cross-Sectional Surveys in Münster, Germany. Int Arch Allergy Immunol. 2005;137:53–61. 15785082 10.1159/000084613

[22] Paulin LM, Williams D'L, Peng R, Diette GB, McCormack MC, et al. 24-h Nitrogen dioxide concentration is associated with cooking behaviors and an increase in rescue medication use in children with asthma. Environ Res. 2017;159:118–23. 28797886 10.1016/j.envres.2017.07.052 PMC5623630

[23] Hansel NN, Breysse PN, McCormack MC, Matsui EC, Curtin-Brosnan J, Williams DL, et al. A Longitudinal Study of Indoor Nitrogen Dioxide Levels and Respiratory Symptoms in Inner-City Children with Asthma. Environ Health Perspect. 2008;116:1428–32. 18941590 10.1289/ehp.11349 PMC2569107

[24] Belanger K, Holford TR, Gent JF, Hill ME, Kezik JM, Leaderer BP. Household Levels of Nitrogen Dioxide and Pediatric Asthma Severity. Epidemiology. 2013;24:320–30. 23337243 10.1097/EDE.0b013e318280e2ac PMC3686297

[25] Kanchongkittiphon W, Mendell MJ, Gaffin JM, Wang G, Phipatanakul W. Indoor Environmental Exposures and Exacerbation of Asthma: An Update to the 2000 Review by the Institute of Medicine. Environ Health Perspect. 2015;123:6–20. 25303775 10.1289/ehp.1307922 PMC4286274

[26] Coker ES, Smit E, Harding AK, Molitor J, Kile ML. A cross sectional analysis of behaviors related to operating gas stoves and pneumonia in U.S. children under the age of 5. BMC Public Health. 2015;15:77. 25648867 10.1186/s12889-015-1425-y PMC4321321

[27] Kile ML, Coker ES, Smit E, Sudakin D, Molitor J, Harding AK. A cross-sectional study of the association between ventilation of gas stoves and chronic respiratory illness in U.S. children enrolled in NHANESIII. Environ Health. 2014;13:71. 25182545 10.1186/1476-069X-13-71 PMC4175218

[28] Holm SM, Balmes J, Gillette D, Hartin K, Seto E, Lindeman D *et al*. Cooking behaviors are related to household particulate matter exposure in children with asthma in the urban East Bay Area of Northern California. *PLoS ONE* 2018; 13. 10.1371/journal.pone.0197199. 10.1371/journal.pone.0197199 PMC5991365 29874253

[29] Sun L, Wallace LA. Residential cooking and use of kitchen ventilation: The impact on exposure. J Air Waste Manag Assoc. 2021;71:830–43. 32970538 10.1080/10962247.2020.1823525

[30] Dobbin NA, Sun L, Wallace L, Kulka R, You H, Shin T, et al. The benefit of kitchen exhaust fan use after cooking - An experimental assessment. Build Environ. 2018;135:286–96.

[31] Singer BC, Delp WW, Price PN, Apte MG. Performance of installed cooking exhaust devices: Performance of installed cooking exhaust devices. Indoor Air. 2012;22:224–34. 22044446 10.1111/j.1600-0668.2011.00756.x

[32] Lunden MM, Delp WW, Singer BC. Capture efficiency of cooking-related fine and ultrafine particles by residential exhaust hoods. Indoor Air. 2015;25:45–58. 24750219 10.1111/ina.12118

[33] Zhao H, Chan WR, Cohn S, Delp WW, Walker IS, Singer BC. Indoor air quality in new and renovated low-income apartments with mechanical ventilation and natural gas cooking in California. Indoor Air. 2021;31:717–29. 33070378 10.1111/ina.12764

[34] Zhao H, Chan WR, Delp WW, Tang H, Walker IS, Singer BC. Factors Impacting Range Hood Use in California Houses and Low-Income Apartments. Int J Environ Res Public Health. 2020;17:8870. 33260667 10.3390/ijerph17238870 PMC7729668

[35] Sun L, Singer BC Cooking methods and kitchen ventilation availability, usage, perceived performance and potential in Canadian homes. *J Expo Sci Environ Epidemiol* 2023. 10.1038/s41370-023-00543-z. 10.1038/s41370-023-00543-z PMC10234804 37059807

[36] Lajoie P, Aubin D, Gingras V, Daigneault P, Ducharme F, Gauvin D, et al. The IVAIRE project - a randomized controlled study of the impact of ventilation on indoor air quality and the respiratory symptoms of asthmatic children in single family homes. Indoor Air. 2015;25:582–97. 25603837 10.1111/ina.12181

[37] Paulin LM, Diette GB, Scott M, McCormack MC, Matsui EC, Curtin‐Brosnan J, et al. Home interventions are effective at decreasing indoor nitrogen dioxide concentrations. Indoor Air. 2014;24:416–24. 24329966 10.1111/ina.12085 PMC4909253

[38] Vera E. A Prevention Agenda for 2020 and Beyond: Why Environmental Interventions Matter Now More Than Ever. J Prev Health Promot. 2020;1:5–33.

[39] Abrham Y, Zeng S, Tenney R, Davidson C, Yao E, Kloth C, et al. Effect of a single one-hour teaching session about environmental pollutants and climate change on the understanding and behavioral choices of adolescents: The BREATHE pilot randomized controlled trial. PLOS ONE. 2023;18:e0291199. 38011223 10.1371/journal.pone.0291199 PMC10681291

[40] *Communicating Science Effectively: A Research Agenda*. National Academies Press: Washington, D.C., 2017 10.17226/23674. 28406600

[41] Bruine de Bruin W, Bostrom A. Assessing what to address in science communication. Proc Natl Acad Sci. 2013;110:14062–8. 23942122 10.1073/pnas.1212729110 PMC3752171

[42] Abu Abed M, Himmel W, Vormfelde S, Koschack J. Video-assisted patient education to modify behavior: A systematic review. Patient Educ Couns. 2014;97:16–22. 25043785 10.1016/j.pec.2014.06.015

[43] Tuong W, Larsen ER, Armstrong AW. Videos to influence: a systematic review of effectiveness of video-based education in modifying health behaviors. J Behav Med. 2014;37:218–33. 23188480 10.1007/s10865-012-9480-7

[44] OGAWA USA. https://ogawausa.com/ (accessed 23 Apr2024).

[45] Singer BC, Chan WR, Kim Y, Offermann FJ, Walker IS. Indoor air quality in California homes with code‐required mechanical ventilation. Indoor Air. 2020;30:885–99. 32304607 10.1111/ina.12676

[46] Antonopoulos CA, Rosenberg SI, Zhao H, Walker IS, Delp WW, Chan WR, et al. Mechanical ventilation and indoor air quality in recently constructed U.S. homes in marine and cold-dry climates. Build Environ. 2023;245:110480.

[47] Singer BC, Hodgson AT, Hotchi T, Kim JJ. Passive measurement of nitrogen oxides to assess traffic-related pollutant exposure for the East Bay Children’s Respiratory Health Study. Atmos Environ. 2004;38:393–403.

[48] Jones CL, Jensen JD, Scherr CL, Brown NR, Christy K, Weaver J. The Health Belief Model as an Explanatory Framework in Communication Research: Exploring Parallel, Serial, and Moderated Mediation. Health Commun. 2015;30:566–76. 25010519 10.1080/10410236.2013.873363 PMC4530978

[49] US EPA National Center for Environmental Assessment RTPN, Sacks J. Integrated Science Assessment (ISA) for Particulate Matter (Final Report, 2019). 2019.https://cfpub.epa.gov/ncea/isa/recordisplay.cfm?deid=347534 (accessed 7 May2020).

[50] World Health Organization (ed.). *Who guidelines for indoor air quality: selected pollutants*. WHO: Copenhagen, 2010. 23741784

[51] Strand V, Svartengren M, Rak S, Barck C, Bylin G. Repeated exposure to an ambient level of NO2 enhances asthmatic response to a nonsymptomatic allergen dose. Eur Respir J. 1998;12:6–12. 9701406 10.1183/09031936.98.12010006

[52] Buonanno G, Morawska L, Stabile L. Particle emission factors during cooking activities. Atmos Environ. 2009;43:3235–42.

[53] Zhai SR, Albritton D. Airborne particles from cooking oils: Emission test and analysis on chemical and health implications. Sustain Cities Soc. 2020;52:101845.

