# Supplementary material for: Measured air quality impacts after teaching parents about cooking ventilation with a video: a pilot study
Source: J Expo Sci Environ Epidemiol. 2024 Nov 9;35(2):223–32. doi: 10.1038/s41370-024-00730-6 (PMC12009736; doi:10.1038/s41370-024-00730-6)

# Supplemental Information

[Tables 2](#_Toc173374270)

[Table S1. Characteristics of All Children Screened 2](#_Toc173374271)

[Table S2. Measured Range Hood Characteristics and Range Hood Use Before and After Intervention 3](#_Toc173374272)

[Table S3. Burner Use Before and After Intervention 4](#_Toc173374273)

[Table S4. Pollutant Concentrations Before and After Intervention 5](#_Toc173374274)

[Table S5. Respiratory Health Measurements in Study Participants Before and After the Intervention 5](#_Toc173374275)

[Table S6. *P* Values Found from Wilcoxon Signed Rank Tests for the Primary Models and all Sensitivity Analyses 6](#_Toc173374276)

[Figures 7](#_Toc173374277)

[Figure S1. Study Enrollment Diagram 7](#_Toc173374278)

[Figure S2. Measured Range Hood Flow 8](#_Toc173374279)

[Figure S3. Measured Range Hood Sound Levels 8](#_Toc173374280)

[Figure S4. Example of Detailed Monitoring Data from a Single Day at a Single Household 9](#_Toc173374281)

[Figure S5. Percentage of Events with *Any* Range Hood Use among Those that had Co-Occurring Pollution Events 10](#_Toc173374282)

[Figure S6. Percentage of Events with >80% Range Hood Use among Those that had Co-Occurring Pollution Events. 10](#_Toc173374283)

[Figure S7. Delay Between Start of Detected Cooking Event and Start of Range Hood Use. 11](#_Toc173374284)

[Figure S8. Mean CO_2_ Peak During Cooking Events 11](#_Toc173374285)

[Figure S9. Mean FEV1 Z Score Before and After Intervention 12](#_Toc173374286)

[Figure S10. Mean FEV1 Z Score at all Measured Time Points (including siblings, n=18) 12](#_Toc173374287)

[Figure S11. Mean FEV1/FVC Z Score Before and After Intervention 13](#_Toc173374288)

[Figure S12. Mean FEV1/FVC Z Score at all Measured Time Points (including siblings, n=18) 13](#_Toc173374289)

[Figure S13. Mean FeNO Before and After Intervention 14](#_Toc173374290)

[Figure S14. Mean FeNO at all Measured Time Points 14](#_Toc173374291)

[Figure S15. Scatter Plot of Total Integrated PM_2.5_ Concentration ( in μg/m^3^) Compared to the FEV1 Z Score 15](#_Toc173374292)

[Figure S16. Scatter Plot of Total Integrated NO_2_ Concentration ( in ppb) Compared to the FEV1 Z Score 15](#_Toc173374293)

[Figure S17. Scatter Plot of Averaged NO_2_ Concentration ( in ppb) Compared to the FEV1 Z Score 16](#_Toc173374294)

[Figure S18. Scatter Plot of Total Integrated PM_2.5_ Concentration ( in μg/m^3^) Compared to the FeNO 16](#_Toc173374295)

[Figure S19. Scatter Plot of the Total Integrated NO_2_ Concentration ( in ppb) Compared to the FeNO 17](#_Toc173374296)

[Figure S20. Scatter Plot of the Averaged NO_2_ ( in ppb) Compared to the FeNO 17](#_Toc173374297)

## Tables

### Table S1. Characteristics of All Children Screened

| Characteristics of Screened Children | |
| --- | --- |
|  | Overall (N=42) |
| Gender |  |
| girl | 22 (52.4%) |
| boy | 19 (45.2%) |
| non-binary | 1 (2.4%) |
| Sex |  |
| female | 23 (54.8%) |
| male | 19 (45.2%) |
| Race/Ethnicity |  |
| Latinx | 14 (33.3%) |
| Non-Latinx White | 17 (40.5%) |
| Other | 11 (26.2%) |
| Age | 8.7 (0.3) |
| BMI | 16.4 (0.7) |
| History of Asthma |  |
| No | 31 (73.8%) |
| Yes | 11 (26.2%) |
| History of Allergies |  |
| No | 31 (73.8%) |
| Yes | 11 (26.2%) |
| History of Eczema |  |
| No | 34 (81.0%) |
| Yes | 8 (19.0%) |
| History of Prematurity |  |
| No | 36 (85.7%) |
| Yes | 6 (14.3%) |

###

### Table S2. Measured Range Hood Characteristics and Range Hood Use Before and After Intervention

###

| Range Hood Characteristics and Use | | |
| --- | --- | --- |
|  | Pre-Intervention Period  Median (IQR) | Post-Intervention Period  Median (IQR) |
| Measured Flow (cubic feet per minute) among homes that did not have range hoods replaced | 278 (227.5, 397.0) | unchanged |
| Measured Flow (cubic feet per minute) among homes that *did* have range hoods replaced | 89 (49.0, 156.0) | 142 (112.5, 197.8) |
| Measured Sound (dBA) among homes that did not have range hoods replaced | 59.6 (57.5, 65.0) | unchanged |
| Measured Sound (dBA) among homes that *did* have range hoods replaced | 61.7 (55.8, 66.7) | 46.8 (44.5, 50.8) |
| Delay from start of cooking event to onset of range hood use (min) | 1.6 (0.0, 8.2) | 1.6 (0.0, 8.1) |
| Percent of cooking events with *any* range hood use | 23.8 (0.0, 100.0) | 54.2 (0.0, 100.0) |
| Percent of cooking events with range hood use for 80% of the time or more | 3.3 (0.0, 66.7) | 3.7 (0.0, 77.8) |
| Percent of cooking events associated with pollution events with *any* range hood use | 23.4 (0.0, 100.0) | 51.5 (0.0, 100.0) |
| Percent of cooking events associated with pollution events with range hood use for 80% of the time or more | 2.0 (0.0, 80.0) | 10.0 (0.0, 84.6) |

###

###

### Table S3. Burner Use Before and After Intervention

| Burner Use | | |
| --- | --- | --- |
|  | Pre-Intervention Period  Median (IQR) | Post-Intervention Period  Median (IQR) |
| Percent of burner use minutes that include use of a front burner | 77.5 (12.9, 93.9) | 43.1 (9.9, 91.9) |
| Percent of burner use minutes that include use of a back burner | 6.4 (0.0, 70.6) | 38.1 (0.0, 88.4) |
| Percent of burner use minutes that include use of the oven | 34.2 (0.0, 78.6) | 27.4 (7.4, 70.4) |
| Percent of burner use minutes that represent use of *only* a back burner | 1.1 (0.0, 70.6) | 23.9 (0.0, 77.2) |

###

###

### Table S4. Pollutant Concentrations Before and After Intervention

| Pollution Concentrations | | |
| --- | --- | --- |
|  | Pre-Intervention Period  Median (IQR) | Post-Intervention Period  Median (IQR) |
| Mean PM_2.5_ Event Peak (µg/m^3^) | 7.1 (0.6, 80.7) | 8.9 (0.0, 39.0) |
| Mean PM_2.5_ Integrated Event Concentration (µg/m^3^)*min | 384.5 (36.8, 2833.5) | 217.8 (0.0, 1167.1) |
| Mean PM_2.5_ Normalized Event Integrated Concentration ((µg/m^3^*min)/burner-min) | 9.1 (0.7, 36.5) | 5.1 (0.0, 29.0) |
| Mean NO_2_ Event Peak (ppb) | 29.5 (8.4, 118.9) | 16.8 (6.1, 119.8) |
| Mean NO_2_ Integrated Event Concentration (ppb)*min | 1226.1 (336.1, 7861.4) | 756.3 (84.0, 4214.7) |
| Mean NO_2_ Normalized Event Integrated Concentration ((ppb*min)/burner-min) | 30.4 (8.4, 101.2) | 16.1 (3.4, 104.9) |
| Mean CO_2_ Event Peak (ppm) | 984.6 (678.8, 2060.6) | 913.7 (559.9, 2334.6) |
| Across the entire study interval: | Pre-Intervention Period  Median (IQR) | Post-Intervention Period  Median (IQR) |
| PM_2.5_ Total Integrated Concentration (µg/m^3^)*min | 10221.8 (274.7, 65169.9) | 4573.2 (0.0, 31045.4) |
| NO_2_ Total Integrated Concentration (ppb)*min (summed over all cooking events) | 50636.6 (3403.6, 180812.4) | 15879.3 (3742.3, 88507.9) |
| NO_2_ Integrated Concentration (ppb) (from passive samplers) | 10.4 (3.5, 47.5) | 9.4 (3.0, 36.1) |

### Table S5. Respiratory Health Measurements in Study Participants Before and After the Intervention

| Respiratory Health Assessments | | | | |
| --- | --- | --- | --- | --- |
|  | Pre-Intervention Period | | Post-Intervention Period | |
|  | Median (range) | Number of children (n) | Median (range) | Number of children (n) |
| Average FeNO (ppb) | 19.7 (8.0, 53.0) | 9 | 22.5 (5.0, 60.7) | 12 |
| Variability in FeNO measurements | 10% (0, 20%) | 9 | 10% (0, 20%) | 12 |
| FEV_1_ Z-score | 0.5 (-3.0, 1.2) | 11 | -0.5 (-2.3, 0.7) | 11 |
| FVC Z-score | 0.7 (-1.6, 1.5) | 11 | -0.3 (-1.7, 0.8) | 11 |
| FEV_1_ / FVC | 0.9 (0.8, 0.9) | 11 | 0.9 (0.7, 0.9) | 11 |
| FEV_1_ / FVC Z-score | -0.5 (-2.2, 0.5) | 11 | -0.6 (-2.7, 0.4) | 11 |
| Childhood ACT Score | 25 (23-26) | 4 | 26 (24-27) | 3 |

###

### Table S6. *P* Values Found from Wilcoxon Signed Rank Tests for the Primary Models and all Sensitivity Analyses

|  | *P*-values | | | |
| --- | --- | --- | --- | --- |
| Measure | Main Model | Excluding those with an additional visit prior to the intervention | Excluding those whose households did not get indicated range hood replacements | Restricting to participants with atopy |
| Cooking events with any range hood use | 0.018* | 0.024* | 0.058 | 0.030* |
| Cooking events with range hood use for greater than or equal to 80% of the event | 0.042* | 0.036* | 0.142 | 0.142 |
| Self-reported range hood use | 0.120 | 0.120 | 0.071 | 0.773 |
| Cooking events associated with pollution with any range hood use | 0.041* | 0.076 | 0.080 | 0.022* |
| Cooking events associated with pollution with range hood use for greater than or equal to 80% of the event | 0.286 | 0.108 | 0.100 | 0.100 |
| Delay in turning on range hood after beginning of the cooking event | 0.824 | 0.683 | 0.944 | 0.554 |
| % of burner minutes that represent use of the front burner | 0.004** | 0.009* | 0.018* | 0.044* |
| % of burner minutes that represent use of the back burner | 0.021* | 0.029* | 0.126 | 0.076 |
| Self-reported burner use in the prior week | 0.011* | 0.020* | 0.037* | 0.037* |
| Mean PM_2.5_ Event Peak | 0.208 | 0.108 | 0.185 | 0.834 |
| Mean PM_2.5_ Integrated Event Concentration | 0.124 | 0.065 | 0.126 | 0.944 |
| Mean PM_2.5_ Normalized Event Integrated Concentration | 0.108 | 0.065 | 0.126 | 0.834 |
| PM_2.5_ Total Integrated Concentration | 0.050 | 0.025* | 0.025* | 0.441 |
| Mean NO_2_ Event Peak | 0.059 | 0.045* | 0.053* | 0.441 |
| Integrated NO_2_ Integrated Event Concentration | 0.025* | 0.013* | 0.041* | 0.363 |
| Mean NO_2_ Normalized Event Integrated Concentration | 0.108 | 0.038* | 0.185 | 0.294 |
| NO_2_ Total Integrated Concentration (during all cooking events) | 0.043* | 0.078 | 0.025* | 0.141 |
| Integrated NO_2_ during the entire measurement interval | 0.002** | 0.003** | 0.008* | 0.014* |
| Mean CO_2_ Event Peak | 0.184 | 0.170 | 0.032* | 0.294 |
| Mean FeNO | 0.058 | 0.107 | 0.076 | 0.106 |
| FEV_1_ Z score | 0.056 | 0.058 | 0.193 | 0.272 |
| FEV_1_ / FVC Z score | 0.894 | 0.726 | 0.726 | 0.272 |

**p* < 0.05, ***p* <0.005

## Figures

### Figure S1. Study Enrollment Diagram


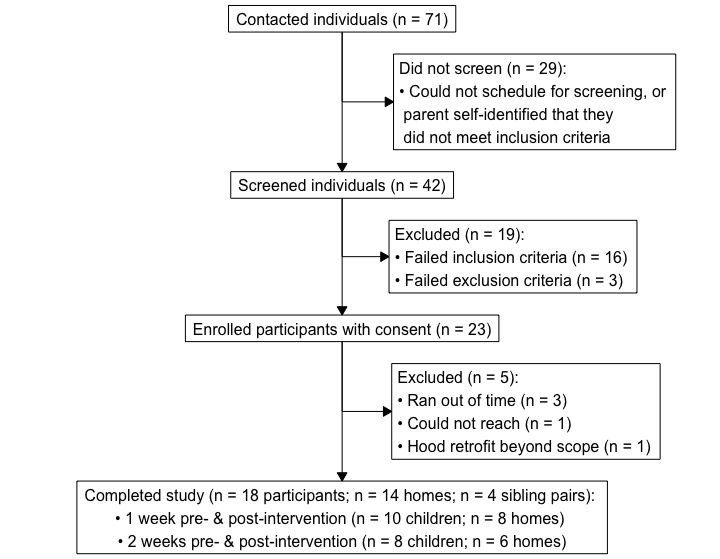


Out of the 14 homes that completed the study, 13 had air pollution data in both pre and post intervention periods.

### Figure S2. Measured Range Hood Flow


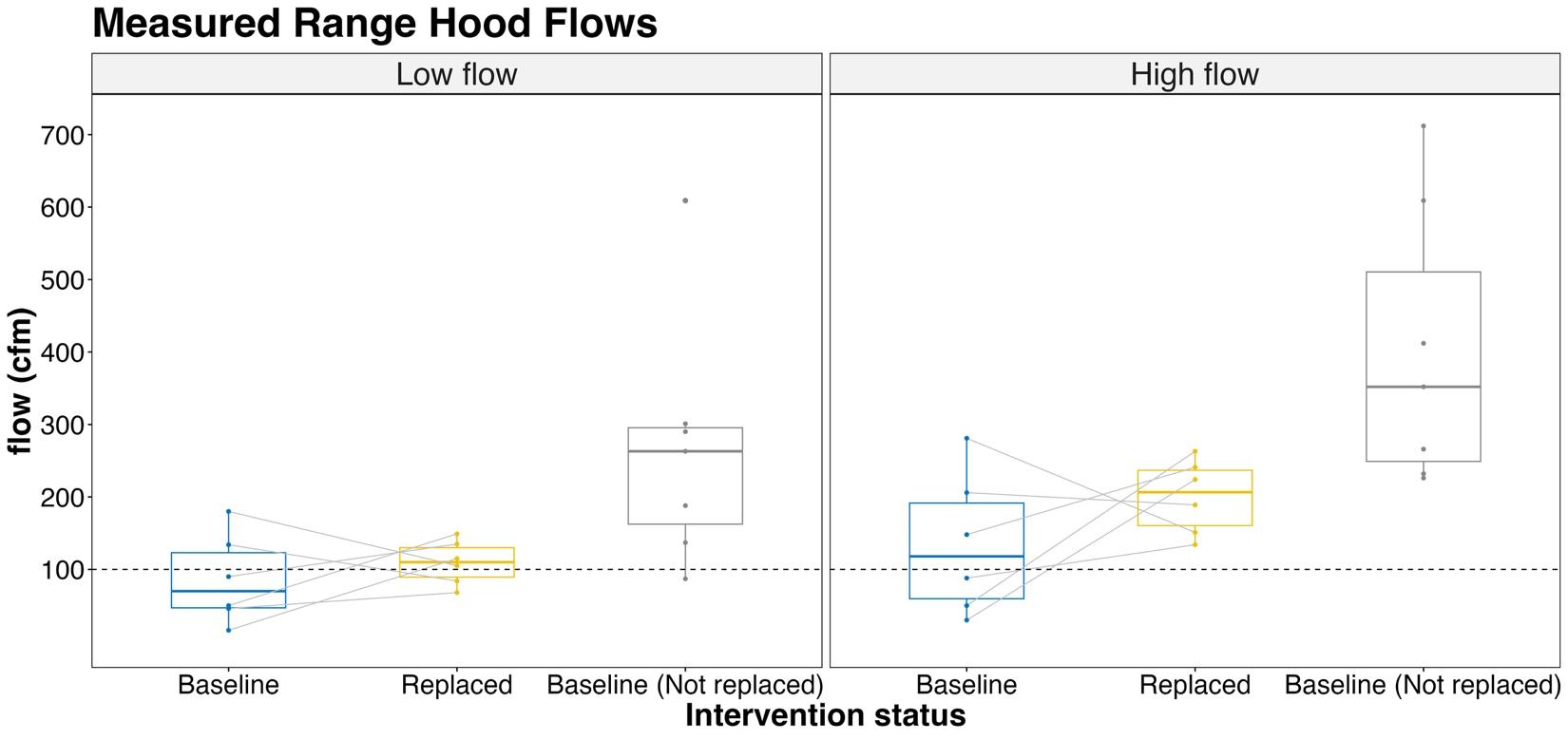


### Figure S3. Measured Range Hood Sound Levels


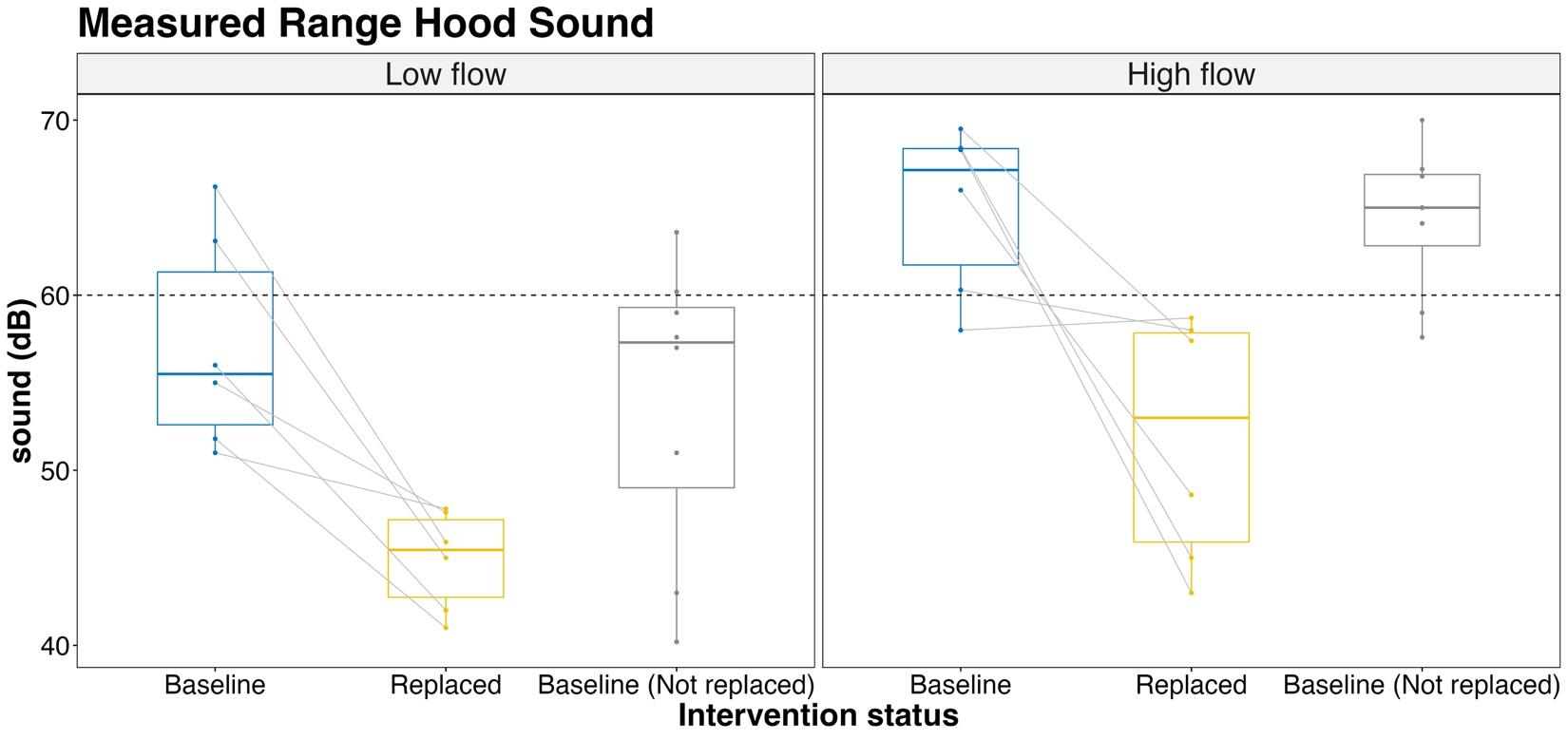


### Figure S4. Example of Detailed Monitoring Data from a Single Day at a Single Household
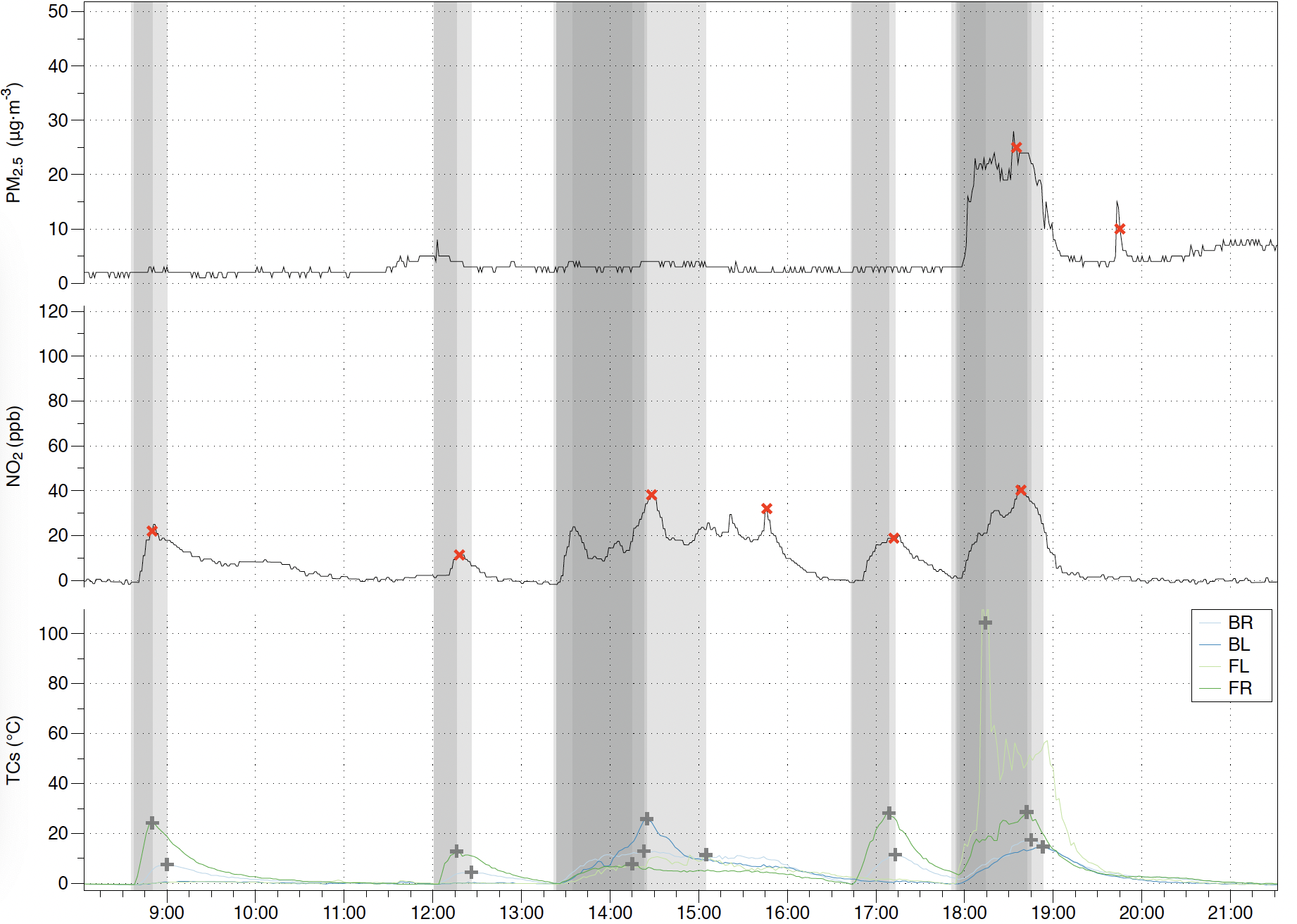


Shown are time-aligned PM_2.5_, NO_2_ and stove temperature data. The temperature graph shows data from 5 thermocouples, one located near each of 4 burners (Back Right (BR), Back Left (BL), Front Left (FL) and Front Right (FR)). Markers (x or +) indicate algorithmically identified peaks in temperature or pollutant concentrations; front burners have green tracings and back burners have blue tracings.

Shaded bars are marking the algorithmically identified start of a temperature “event,” through the peak temperature at that thermocouple. The shades are darker when multiple thermocouples had temperature events simultaneously. Clear NO_2_ peaks coincide with many temperature peaks (or in the case of the example plot above, with all identified temperature peaks). Some temperature peaks are also associated with PM_2.5_ peaks, though there are unrelated PM_2.5_ peaks as well.

Examples captured in this graph include cooking events around 8:40 am, noon and 5 pm, during which the front right burner was used to cook and the thermocouple for the back right burner picked up some heat “shadow” from the front burner, meaning that it has a demonstrated peak that is slightly delayed from the front burner but following the same shape. Around 2p, there was a more complicated cooking event that likely included use of the oven (slow steady increase in the temperature near most burners simultaneously) plus use of a single burner, and around 6p there was a two-burner cooking event (likely with some temperature adjustments made at multiple time points in the front left burner).

### Figure S5. Percentage of Events with *Any* Range Hood Use among Those that had Co-Occurring Pollution Events


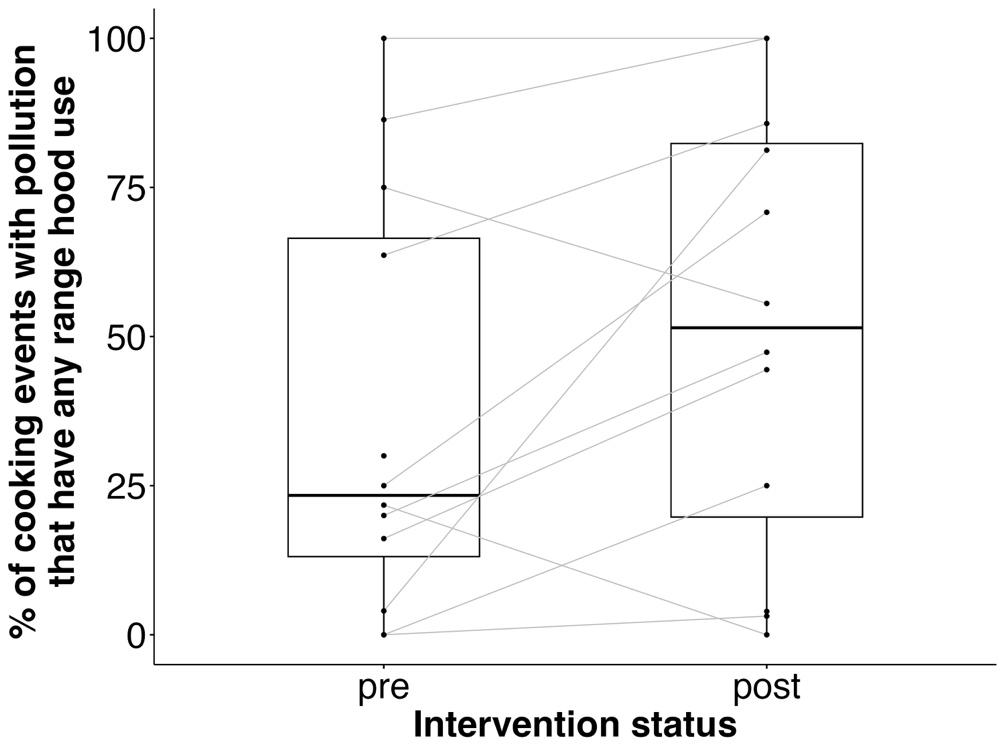


### Figure S6. Percentage of Events with >80% Range Hood Use among Those that had Co-Occurring Pollution Events.


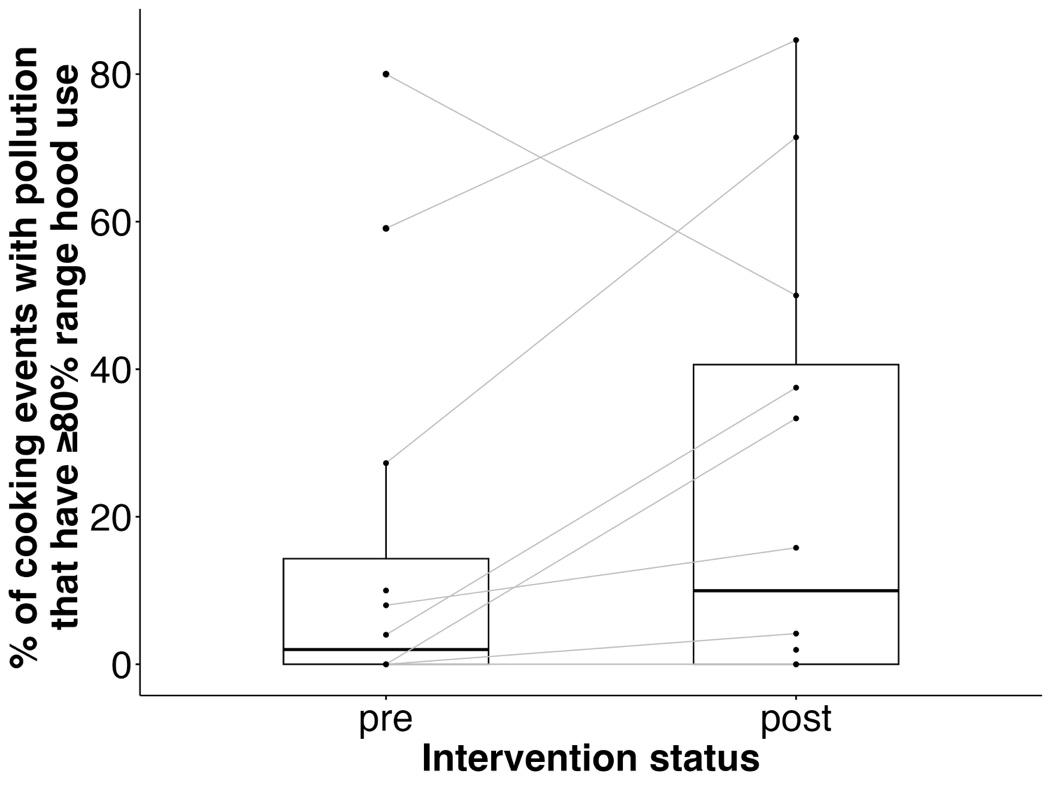


### Figure S7. Delay Between Start of Detected Cooking Event and Start of Range Hood Use.


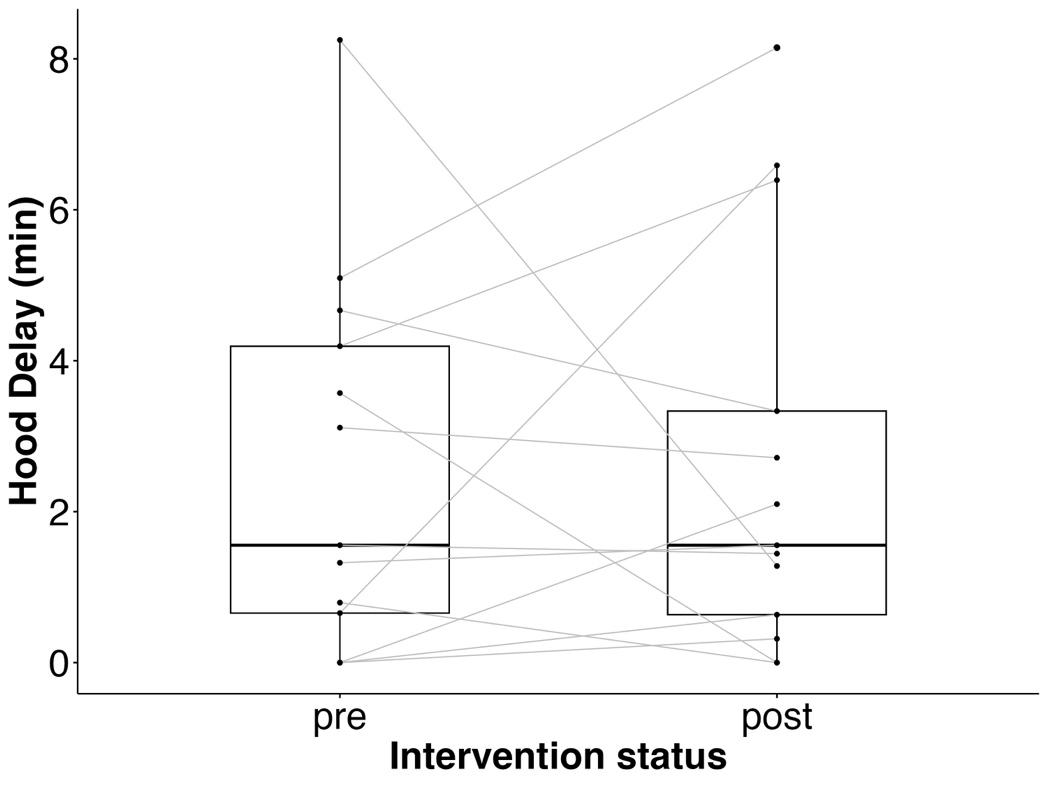


### Figure S8. Mean CO_2_ Peak During Cooking Events


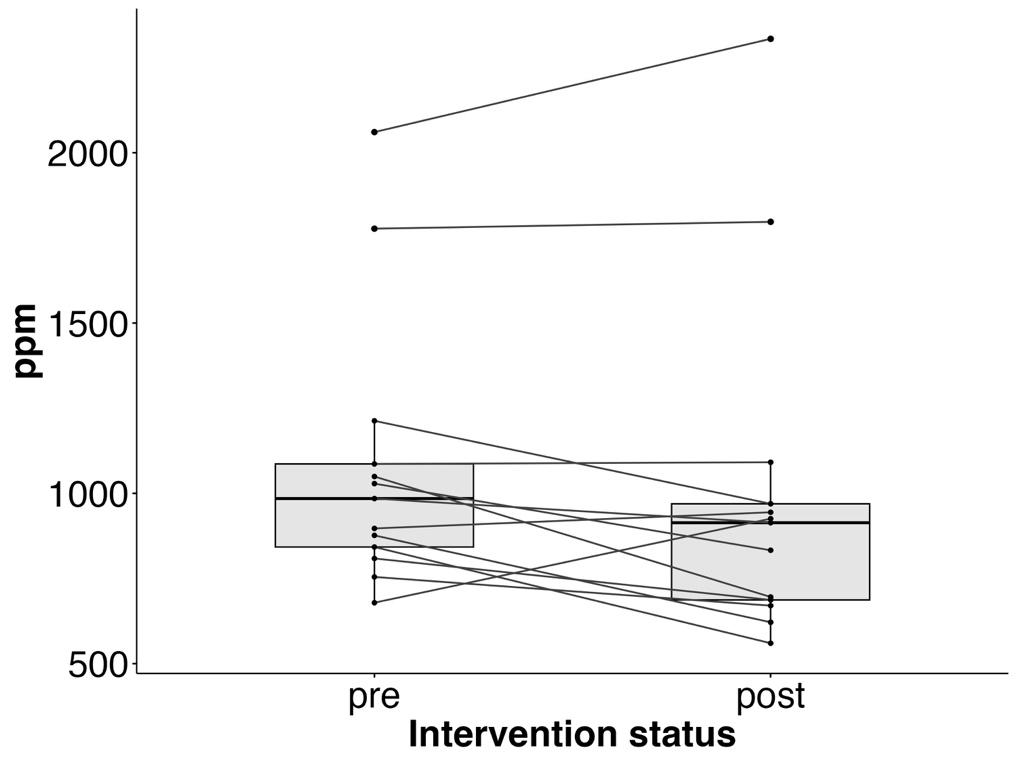


### Figure S9. Mean FEV1 Z Score Before and After Intervention


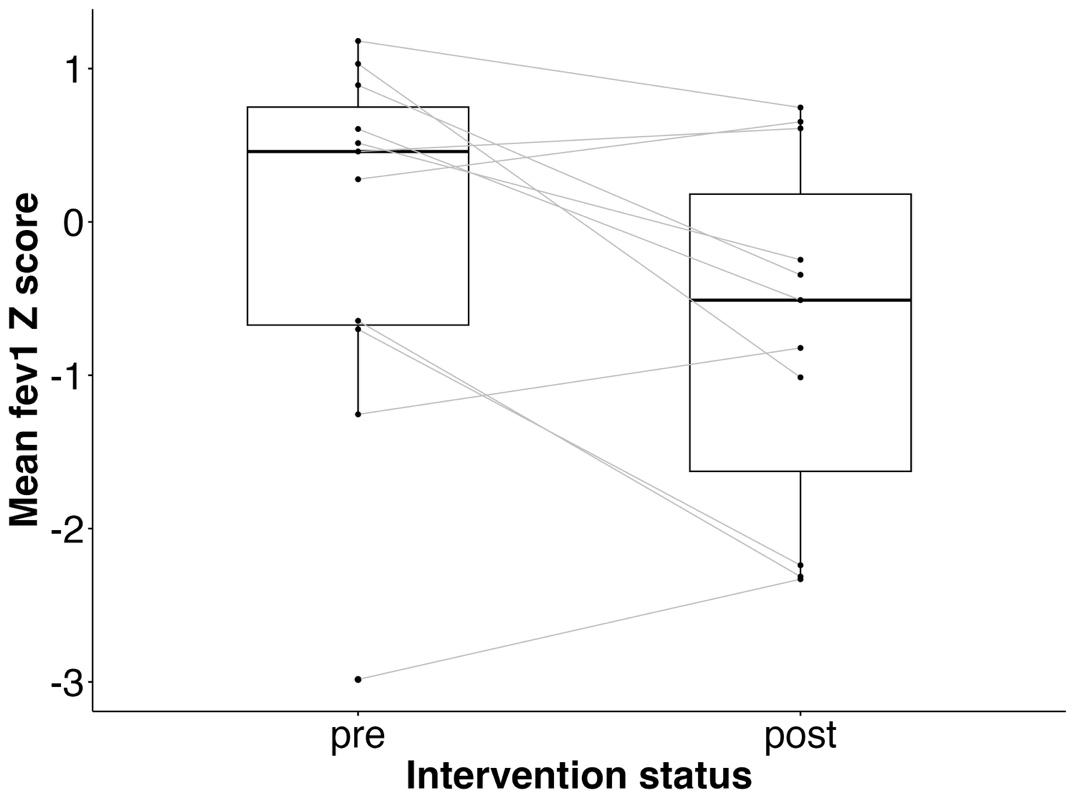


### Figure S10. Mean FEV1 Z Score at all Measured Time Points (including siblings, n=18)


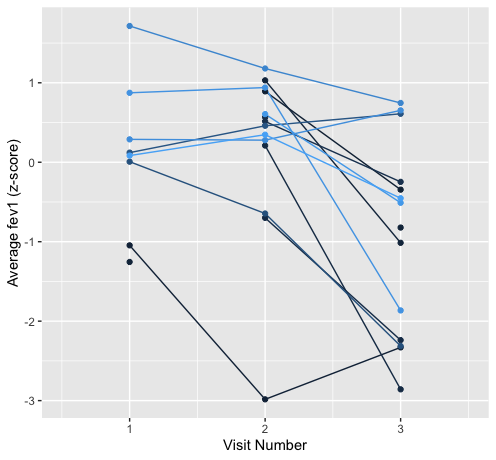


### Figure S11. Mean FEV1/FVC Z Score Before and After Intervention


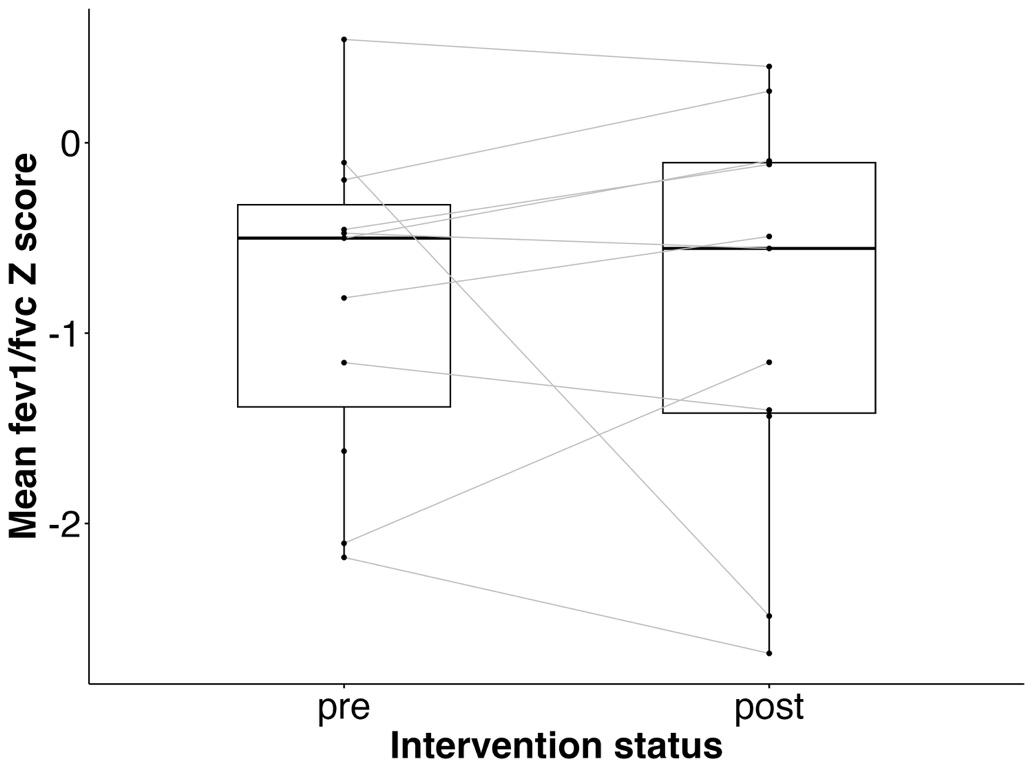


### Figure S12. Mean FEV1/FVC Z Score at all Measured Time Points (including siblings, n=18)


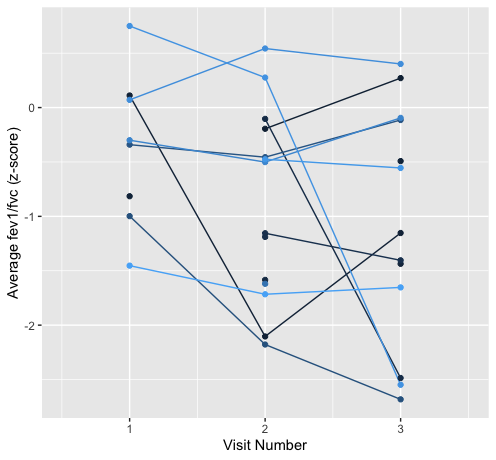


### Figure S13. Mean FeNO Before and After Intervention


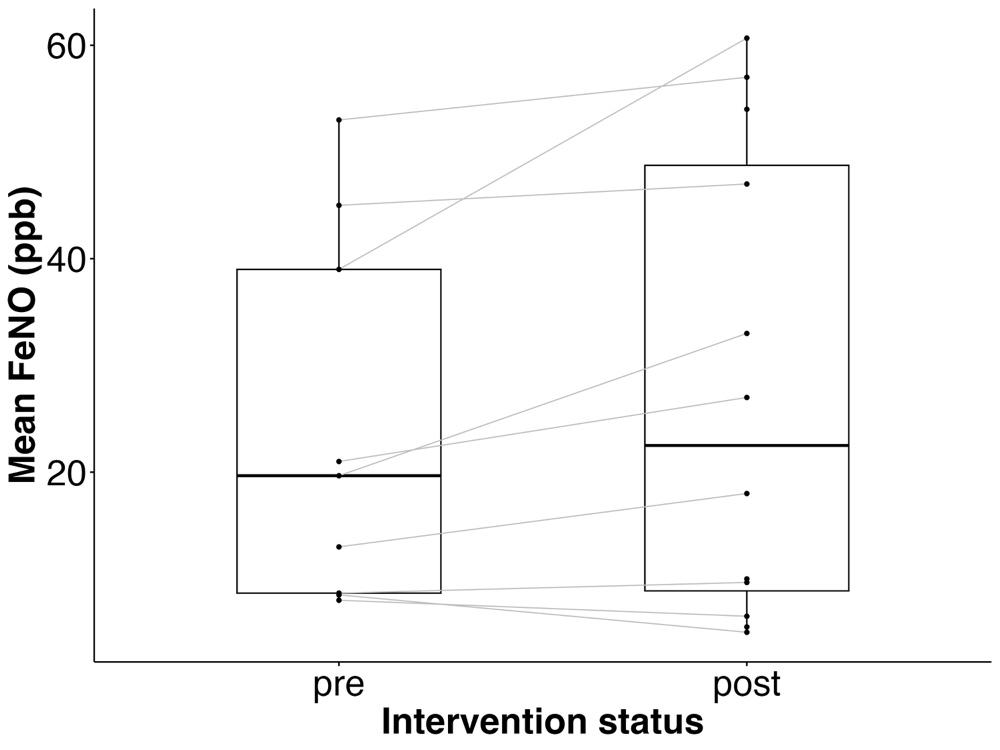


### Figure S14. Mean FeNO at all Measured Time Points

Mean FeNO at all measured time points (including siblings, n=18). Intervention occurred between visits 2-3, so the pre-post analysis was of those two visits.


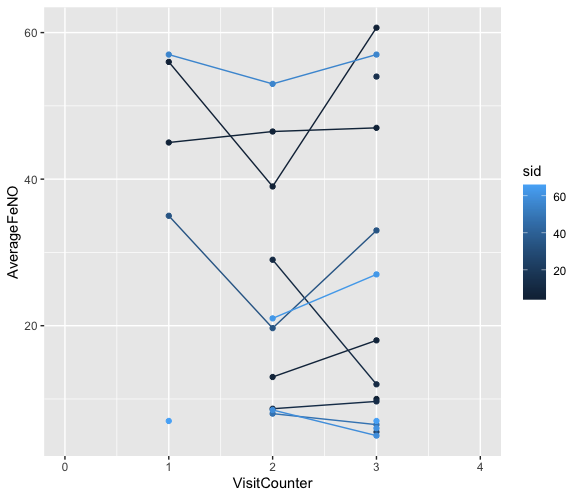


### Figure S15. Scatter Plot of Total Integrated PM_2.5_ Concentration ( in μg/m^3^) Compared to the FEV1 Z Score

Scatter plot of the total integrated PM_2.5_ Concentration for that intervention phase (before or after), compared to the FEV1 Z score measured for that participant (n=14, one per household). Lines connect measurement points from the same participant.


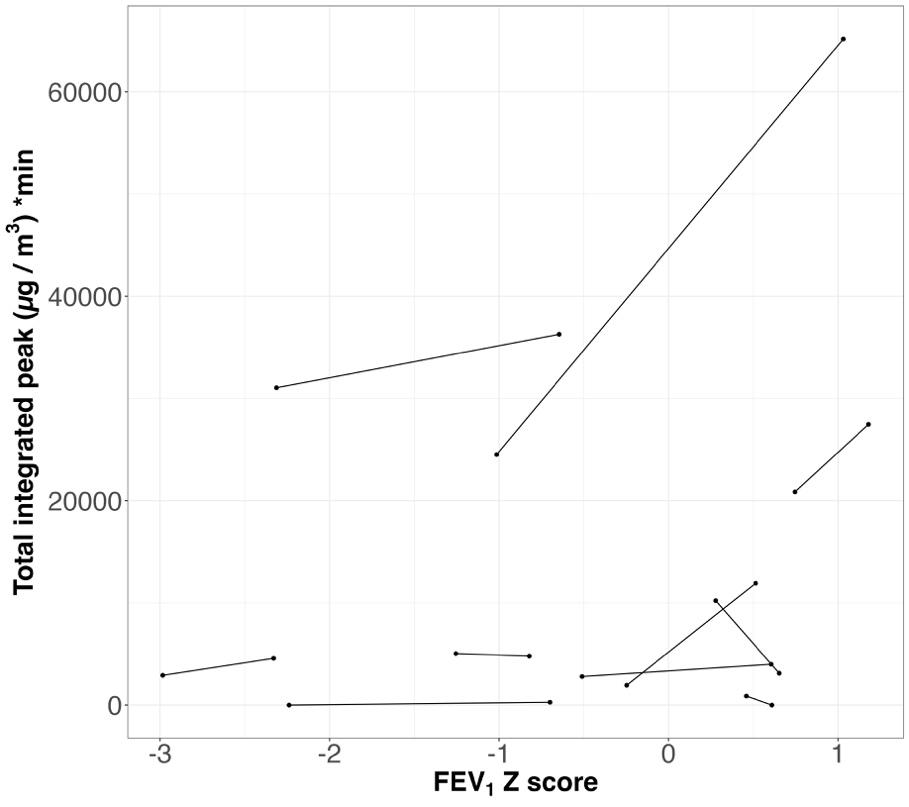


### Figure S16. Scatter Plot of Total Integrated NO_2_ Concentration ( in ppb) Compared to the FEV1 Z Score

Scatter plot of the total integrated NO_2_ Concentration for that intervention phase (before or after), compared to the FEV_1_ Z score measured for that participant (n=14, one per household). Lines connect measurement points from the same participant.


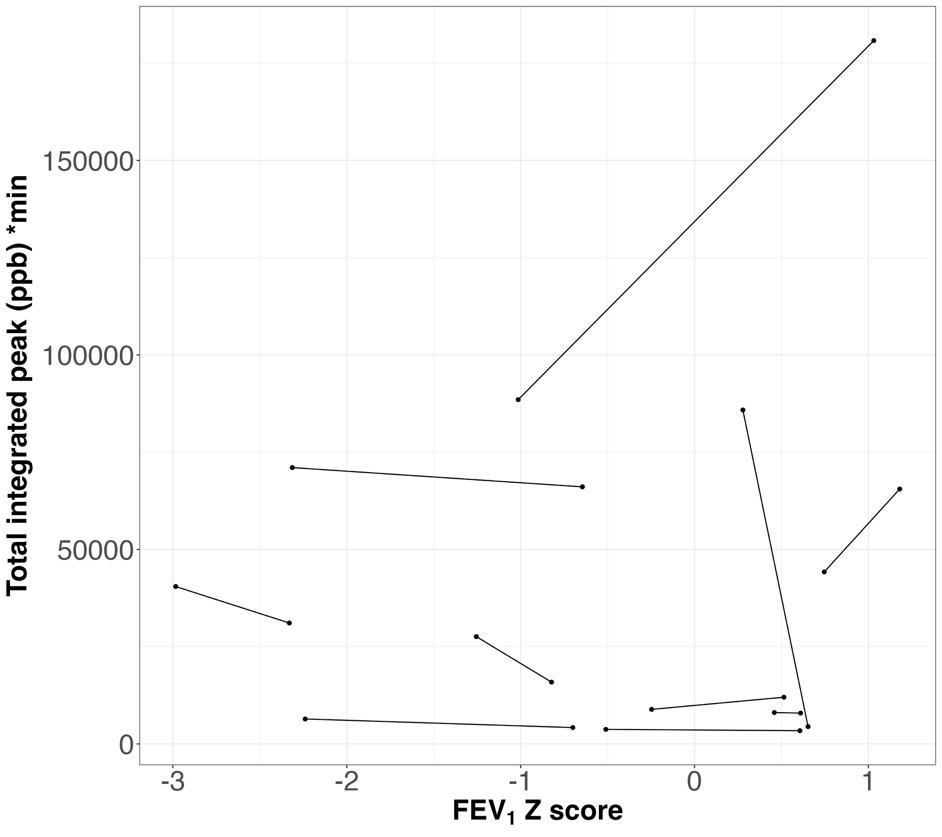


### Figure S17. Scatter Plot of Averaged NO_2_ Concentration ( in ppb) Compared to the FEV1 Z Score

Scatter plot of the averaged NO_2_ for that intervention phase (before or after, not restricted to cooking intervals), compared to the FEV_1_ Z score measured for that participant (n=14, one per household). Lines connect measurement points from the same participant.


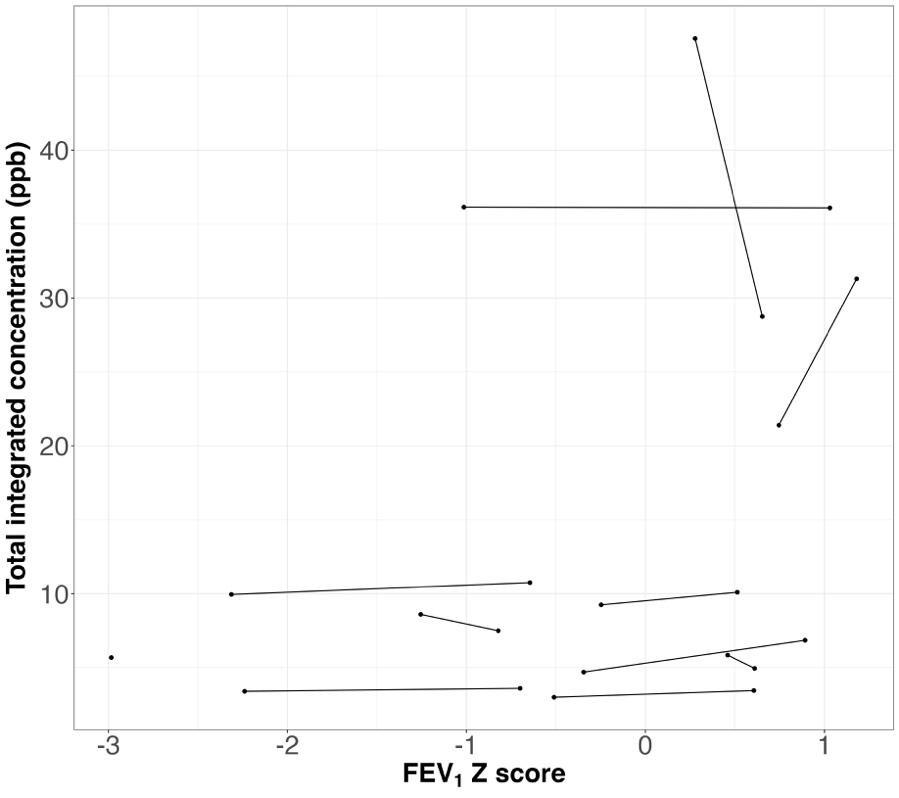


### Figure S18. Scatter Plot of Total Integrated PM_2.5_ Concentration ( in μg/m^3^) Compared to the FeNO

Scatter plot of the total integrated PM_2.5_ Concentration for that intervention phase (before or after), compared to the FeNO measured for that participant (n=14, one per household). Lines connect measurement points from the same participant.


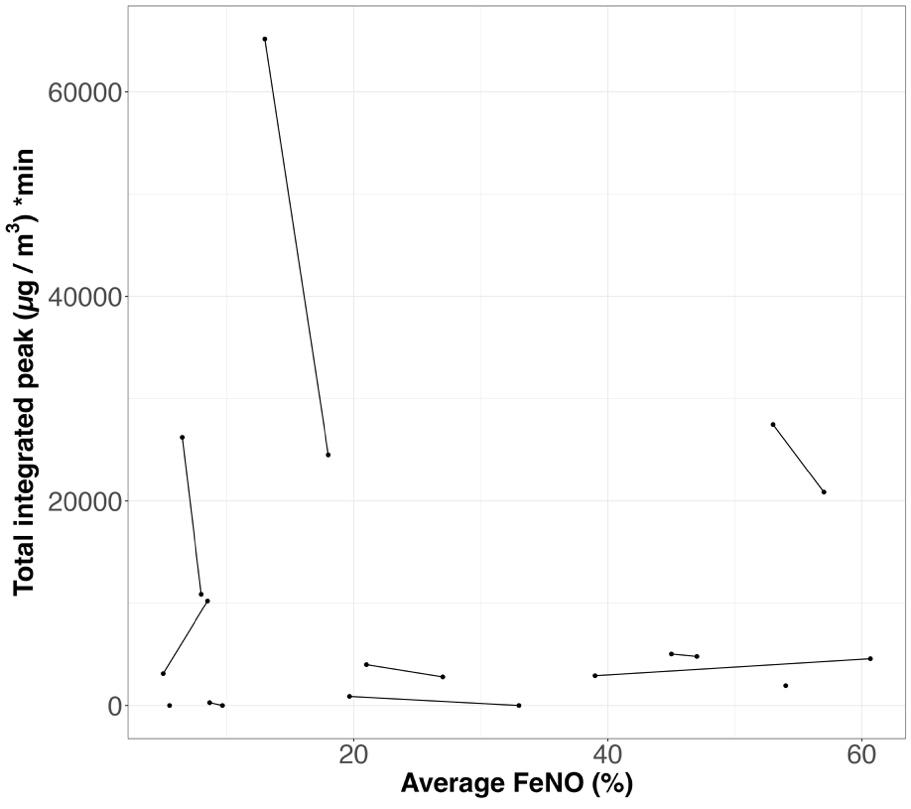


### Figure S19. Scatter Plot of the Total Integrated NO_2_ Concentration ( in ppb) Compared to the FeNO

Scatter plot of the total integrated NO_2_ Concentration for that intervention phase (before or after) compared to the FeNO measured for that participant (n=14, one per household). Lines connect measurement points from the same participant.


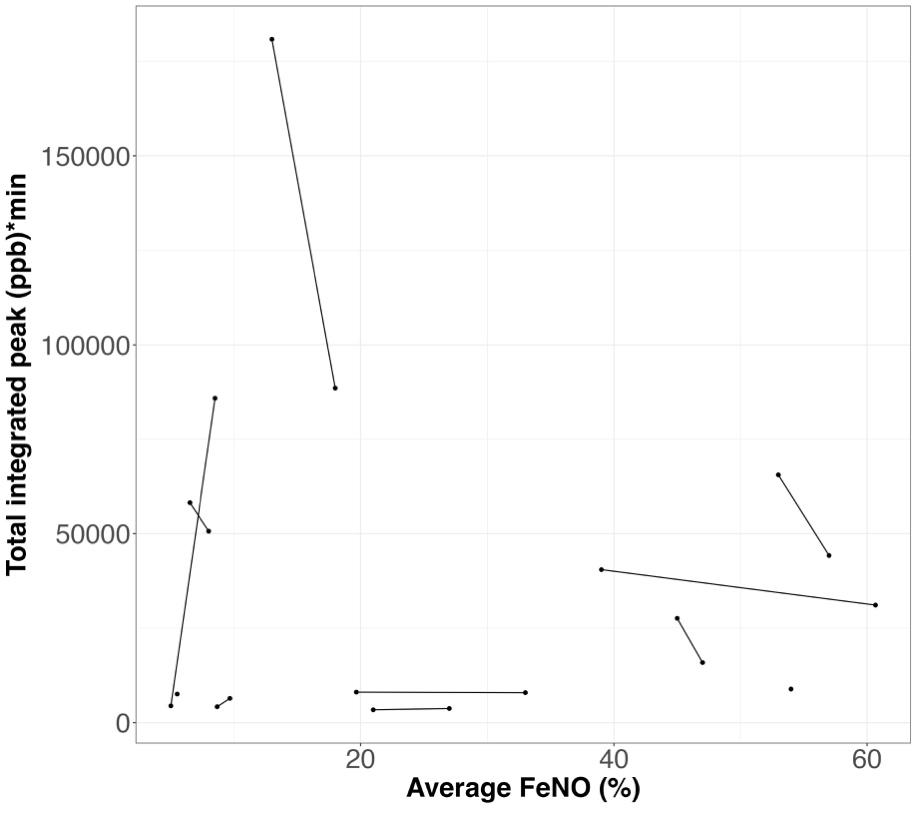


### Figure S20. Scatter Plot of the Averaged NO_2_ ( in ppb) Compared to the FeNO

Scatter plot of the averaged NO_2_ for that intervention phase (before or after, not restricting to cooking intervals), compared to the FeNO measured for that participant (n=14, one per household). Lines connect measurement points from the same participant.


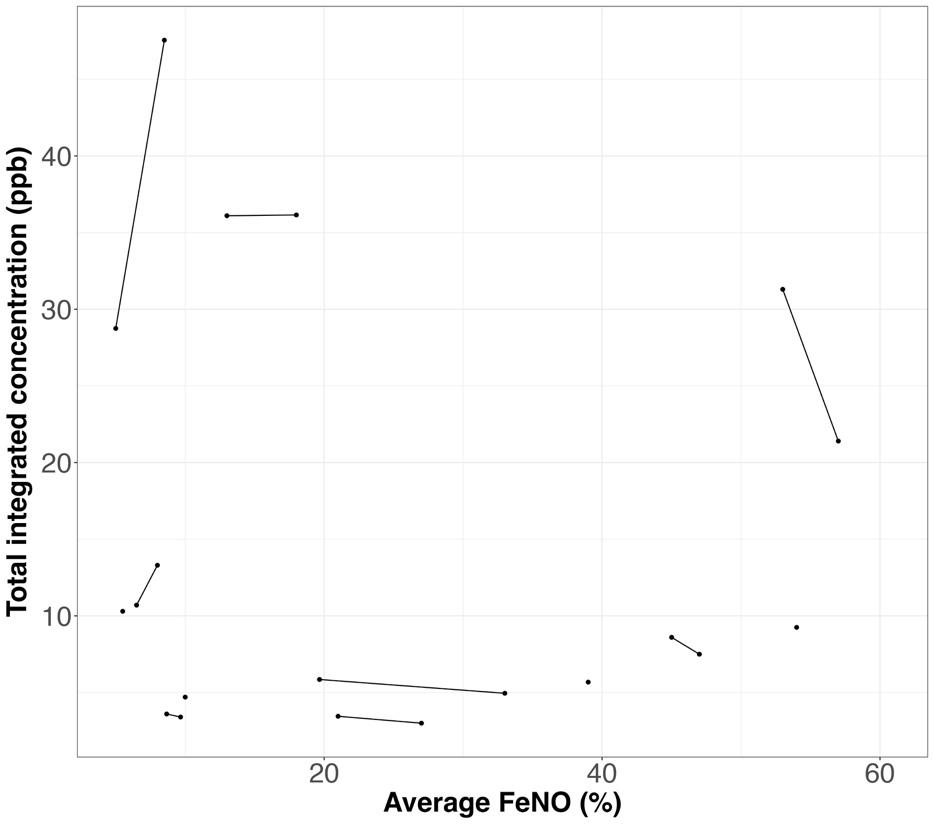

Supplement: Supplementary file 1 — Supplemental information [file 41370_2024_730_MOESM1_ESM.docx]
